# Disruption of G-quadruplex dynamicity by BRCA2 abrogation instigates phase separation and break-induced replication at telomeres

**DOI:** 10.1093/nar/gkae251

**Published:** 2024-04-08

**Authors:** Jennifer J Lee, Hyungmin Kim, Haemin Park, UkJin Lee, Chaelim Kim, Min Lee, Yongdae Shin, Ji-Jung Jung, Han-Byoel Lee, Wonshik Han, Hyunsook Lee

**Affiliations:** Department of Biological Sciences & Institute of Molecular Biology and Genetics (IMBG), Seoul National University, 1 Gwanak-Ro, Gwanak-Gu, Seoul 08826, Korea; Department of Biological Sciences & Institute of Molecular Biology and Genetics (IMBG), Seoul National University, 1 Gwanak-Ro, Gwanak-Gu, Seoul 08826, Korea; Department of Biological Sciences & Institute of Molecular Biology and Genetics (IMBG), Seoul National University, 1 Gwanak-Ro, Gwanak-Gu, Seoul 08826, Korea; Department of Biological Sciences & Institute of Molecular Biology and Genetics (IMBG), Seoul National University, 1 Gwanak-Ro, Gwanak-Gu, Seoul 08826, Korea; Interdisciplinary Program in Bioengineering, Seoul National University, Seoul 08826, Korea; Interdisciplinary Program in Bioengineering, Seoul National University, Seoul 08826, Korea; Department of Mechanical Engineering, Seoul National University, Seoul 08826, Korea; Interdisciplinary Program in Bioengineering, Seoul National University, Seoul 08826, Korea; Department of Surgery, Seoul National University College of Medicine, Seoul 03080, Korea; Department of Surgery, Seoul National University College of Medicine, Seoul 03080, Korea; Biomedical Research Institute, Seoul National University Hospital, Seoul 03080, Korea; Cancer Research Institute, Seoul National University, Seoul 03080, Korea; Department of Surgery, Seoul National University College of Medicine, Seoul 03080, Korea; Biomedical Research Institute, Seoul National University Hospital, Seoul 03080, Korea; Cancer Research Institute, Seoul National University, Seoul 03080, Korea; Department of Biological Sciences & Institute of Molecular Biology and Genetics (IMBG), Seoul National University, 1 Gwanak-Ro, Gwanak-Gu, Seoul 08826, Korea

## Abstract

Dynamic interaction between BRCA2 and telomeric G-quadruplexes (G4) is crucial for maintaining telomere replication homeostasis. Cells lacking BRCA2 display telomeric damage with a subset of these cells bypassing senescence to initiate break-induced replication (BIR) for telomere synthesis. Here we show that the abnormal stabilization of telomeric G4 following BRCA2 depletion leads to telomeric repeat-containing RNA (TERRA)-R-loop accumulation, triggering liquid–liquid phase separation (LLPS) and the assembly of Alternative Lengthening of Telomeres (ALT)-associated promyelocytic leukemia (PML) bodies (APBs). Disruption of R-loops abolishes LLPS and impairs telomere synthesis. Artificial engineering of telomeric LLPS restores telomere synthesis, underscoring the critical role of LLPS in ALT. TERRA-R-loops also recruit Polycomb Repressive Complex 2 (PRC2), leading to tri-methylation of Lys27 on histone H3 (H3K27me3) at telomeres. Half of paraffin-embedded tissue sections from human breast cancers exhibit APBs and telomere length heterogeneity, suggesting that *BRCA2* mutations can predispose individuals to ALT-type tumorigenesis. Overall, BRCA2 abrogation disrupts the dynamicity of telomeric G4, producing TERRA-R-loops, finally leading to the assembly of telomeric liquid condensates crucial for ALT. We propose that modulating the dynamicity of telomeric G4 and targeting TERRA-R-loops in telomeric LLPS maintenance may represent effective therapeutic strategies for treating ALT-like cancers with APBs, including those with *BRCA2* disruptions.

## Graphical Abstract

**Graphical Abstract:**
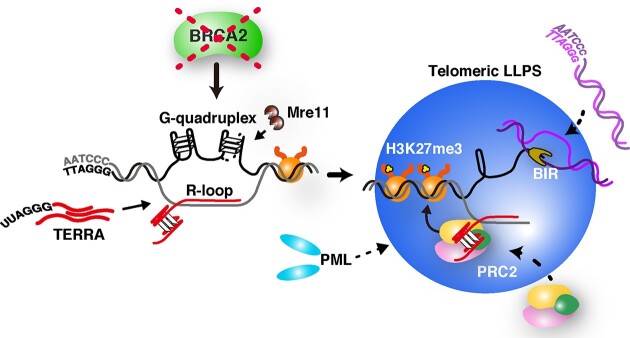

## Introduction

Telomeres are highly organized structures at the end of linear eukaryotic chromosomes comprised of long repetitive guanine-rich sequences. Critical functions of telomeres include restraining uncontrolled cell division and maintaining genome integrity. Every time eukaryotic cells divide, telomeres shorten, eventually leading to cellular senescence. Indeed, for cancer cells to achieve immortality in continuous replication, telomere shortening must be overcome. The majority of cancer cells achieve this goal by reactivating telomerase activity. However, 10–15% of cancer cells progress along an alternative path, the alternative lengthening of telomeres (ALT). ALT utilizes DNA repair mechanisms, such as break-induced replication (BIR) (1), an error-prone conservative DNA synthesis mechanism, or homologous recombination (HR) (2) to overcome telomere shortening during tumorigenesis.

The tumor suppressor BRCA2 is critically required for the maintenance of genome integrity throughout the cell cycle (3). Importantly, BRCA2 is involved in telomere replication homeostasis (4,5): lack of BRCA2 increases telomere shortening, particularly on the lagging strand (5). Paradoxically, the absence of Brca2 in telomerase-deficient mouse fibroblasts (6) or *Caenorhabditis elegans* (7) instigates BIR at telomeres. The absence of BRCA2 selectively chooses BIR over HR for the repair of telomere damage (8). However, the molecular mechanism underlying what triggers ALT remains unclear.

We recently reported that BRCA2 recognizes dynamically interconverting telomere G-quadruplex (G4), a G-rich four-stranded nucleic acid structure. Specifically, we demonstrated that BRCA2 directly binds to G-triplex (G3) intermediates during dynamic interconversion between parallel and non-parallel telomeric G4s (9). The binding of BRCA2 to G3 protects telomeric G4, which would otherwise be susceptible to attack by nuclease MRE11. At the same time, BRCA2 binding to G3 enables RAD51 loading to single-stranded regions in G3, facilitating the remodeling of the stalled replication fork and restarting telomere replication (9).

ALT cancer cells often contain ALT-associated promyelocytic leukemia bodies (APBs) (10). Promyelocytic leukemia (PML) protein is known to drive liquid–liquid phase separation (LLPS), leasding to the membrane-less organelle, by helping multivalent protein interactions (11,12). Min *et al.* artificially generated telomere clusters by overexpressing nuclear polySUMO (small ubiquitin-like modification)/polySIM (SUMO-interacting motif) condensates and showed that it possesses a phase separation property (13,14). When BLM helicase and RAD52 were present in the artificially generated telomere droplet, BIR-mediated telomere synthesis occurred (15). Depletion of PML protein failed to maintain telomere length in ALT cells (16–18). However, what triggers APB assembly and how ALT is associated remains elusive.

Previously, we showed that BRCA2 deficiency results in the loss of G4 dynamics, leading to replication fork stalling and an increase in resection by MRE11 during telomere lagging strand synthesis (9). We now extend these findings substantially and show that abnormally stabilized telomeric G4 in cells lacking BRCA2 induces transcription of telomere repeat transcript, TERRA, subsequently leading to the accumulation of DNA:RNA hybrid R-loops at telomeres. We show that TERRA-containing R-loops trigger LLPS and telomere synthesis. Depletion of any component of telomere LLPS abolished phase separation as well as telomere synthesis activity, suggesting that the components of telomere LLPS form a tight functional interaction network and are interdependent on each other for the maintenance of liquid condensate. We suggest that modulating the dynamicity of telomere G4 or the TERRA-R-loop, or interfering with the dense property of telomere LLPS may have clinical implications for treating APB-positive ALT cancers, including *BRCA2* deficiency-induced ALT-like cancers.

## Materials and methods

### Statistical analysis

GraphPad Prism 5 software was used for drawing all the graphs and statistical analysis. The Student's *t*-test was used for calculating *P*-values, unless stated otherwise. The mean ± standard error of the mean (SEM) is shown when appropriate.

### Mouse breeding, immortalization of MEFs to generate TBI fibroblasts, and conditional depletion of *Brca2*

Mouse breeding and isolation of third-generation *Brca2^F11/F11^; mTR^−/−^; Cre-ER™* mouse embryo fibroblasts (MEFs) followed previously described methods (6), as did the generation and use of TBI fibroblasts [telomerase-null (*mTR^−/−^*); *Brca2^F11/F11^*; *Cre-ER™*] (9). Mouse experiments were approved by the Institutional Animal Care and Use of Committee (IACUC) of Seoul National University (SNU-200323-7-1). We strictly followed Seoul National University guidelines, policies and regulations for the Care and Use of Laboratory Animals. *Brca2*-conditional and *mTR*-knockout (KO) MEFs were immortalized through retroviral infection of pBABE-zeo largeTgenomic (Addgene # 1778), followed by selection with zeocin for 2 weeks. To delete the *Brca2* allele, immortalized MEFs were treated with 1 μM 4-hydroxytamoxifen (4-OHT).

### Cell culture, transfection and generation of stable cell lines

HeLa LT *TERC* KO and BJ cells were provided by Jaewon Min (19). Human cells were grown in Dulbecco's modified Eagle's medium (DMEM) supplemented with 10% fetal bovine serum (FBS) at 37°C with 5% CO_2_. Small interfering RNAs (siRNAs) and plasmids were transfected for 3 days. Knock-down or protein overexpression was confirmed by western blot analysis.

To generate lentiviral particles, lentiviral packaging plasmid psPAX2 (Addgene # 12260), envelope plasmid pMD2.G (Addgene # 12259) and lentiviral constructs were co-transfected into 293FT cells. After 3 days, lentiviral supernatant was collected and concentrated, and target cells were infected in the presence of 4 μg/ml polybrene (hexadimethrine bromide, Sigma-Aldrich, H9268).

### Plasmids and siRNAs

LLPS-inducing constructs were cloned into the pHR-SFFV vector. The RNH1–green fluorescent protein (GFP) vector was purchased from Sino Biological (MG52212-ACGLN). Full-length BRCA2 was cloned into pEGFP-C1 vector. siRNA resistance was achieved through generating silent mutations (5′-GAG GAG CAG TAC CCA ACC A-3′) against the siBRCA2 target sequence (5′-GAA GAA CAA TAT CCT ACT A-3′). siRNAs used in this study include: siLuciferase (SP-3003, Bioneer); mouse siPML (20); human siPML (21); human siBRCA2 (22); siEZH2#1, 5′-CAUUGGUACUUACUACGAU-3′ (SASI_MM01_00061987, Sigma-Aldrich); siEZH2#2, 5′-GAGCAAAGCUUGCAUUCAU-3′ (SASI_MM01_00061985, Sigma-Aldrich); siSUZ12 (23); siEED (24); and human siEZH2 (25).

### Antibodies

Anti-Brca2 antibody was generated in-house (22). The following antibodies were purchased: anti-PML (MAB3738; Merck Millipore), anti-PML [EPR16792] (ab179466; Abcam), anti-PML [PG-M3] (sc-966; Santa Cruz Biotechnology), anti-mCherry [1C51] (ab125096; Abcam), anti-mCherry (ab167453; Abcam), anti-DNA G-quadruplex structures [BG4] (MABE917; Merck Millipore), anti-DYKDDDDK Tag [D6W5B] (#14793; Cell Signaling Technology), anti-DNA–RNA hybrid [S9.6] (MABE1095; Merck Millipore), anti-POLD3 (21935–1-AP; Proteintech), anti-BLM (A300-110A; Bethyl Laboratories), anti-RNase H1 [N2C3] (GTX117624; GeneTex), anti-BRCA2 [AB-1] (OP95; Merck Millipore), anti-H3K27me3 [C36B11] (#9733; Cell Signaling Technology), anti-H3K9me3 (ab8898; Abcam), anti-histone H3 (ab1797; Abcam), anti-EZH2 [D2C9] (#5246; Cell Signaling Technology), anti-SUZ12 (ab12073; Abcam), anti-EED [EPR23043-5] (ab240650; Abcam), anti-vinculin (sc-73614; Santa-Cruz Biotechnology), and anti-beta actin (ab8226; Abcam).

### Quantitative RT–PCR

RNA was extracted by TRIzol™ (15596026, Invitrogen). A 50 ng aliquot of RNA was subjected to one-step quantitative reverse transcription–PCR (RT–qPCR) using a Luna Universal One-step RT–qPCR kit (E3005, New England BioLabs) according to the manufacturer's protocol. The following primers were used to detect mouse mRNA: glyceraldehyde phosphate dehydrogenase (GAPDH) with sense 5′-CCATCAACGACCCCTTCATTGACC-3′ and antisense 5′-TGGTTCACACCCATCACAAACATG-3′; PML with sense 5′-CAGAAAGTCCAGCTGCTCAC-3′ and antisense 5′-GGCACTGTTTTTACCATAGCC-3′; interferon-α with sense 5′-GGACTTTGGATTCCCGCAGGAGAAG-3′ and antisense 5′-GCTGCATCAGACAGCCTTGCAGGTC-3′; interferon-β with sense 5′-TCCGAGCAGAGATCTTCAGGAA-3′ and antisense 5′-TGCAACCACCACTCATTCTGAG-3′; interferon-γ with sense 5′-GAAGTTCTGGGCTTCTCCTCC-3′ and antisense 5′-CTTTTCTTCCACATCTATGCCAC-3′; ISG15 with sense 5′-GGTGTCCGTGACTAACTCCAT-3′ and antisense 5′-TGGAAAGGGTAAGACCGTCCT-3′.

### RNA dot-blot

Total RNA was extracted by the RNeasy Plus Kit (74134, Qiagen) according to the manufacturer's protocol. RNA was dot-blotted on Amersham Hybond™-N^+^ membrane (RPN203B, Cytiva) and hybridized with (CCCTAA)_4_ for TERRA detection, and with GAPDH (5′-TGGTTCACACCCATCACAAACATG-3′) and 18S rRNA (5′-CCATCCAATCGGTAGTAGCG-3′) for the loading and RNA control, respectively. The results are obtained by Typhoon™ FLA 7000 (GE Healthcare Life Sciences).

### Immunofluorescence, telomere-fluorescence *in situ* hybridization (T-FISH) and TERRA RNA-FISH

Cells were pre-extracted with ice-cold CSK buffer [10 mM PIPES pH 8.0, 100 mM NaCl, 300 mM sucrose, 3 mM MgCl_2_, 0.5% (v/v) Triton X-100], then fixed with 4% paraformaldehyde for 10 min. The coverslips were blocked with goat serum, then treated with the indicated primary antibody at 4°C overnight and with secondary antibody at room temperature for 2 h. For staining G4, cells were treated with BG4 antibody at 4°C overnight, followed by anti-FLAG antibody, then anti-rabbit secondary antibody. For T-FISH, cells were dehydrated in 70, 95 and 100% ethanol. After air drying, TelC PNA probes (F1002-5; F1009-5, PANAGENE) were hybridized by boiling at 80°C for 15 min. For TERRA RNA-FISH, TelC PNA probes were hybridized at 37°C for 1 h. For telomere length measurements, cells were synchronized with 800 ng/ml Colcemid and incubated in 0.075 M KCl. Then, cells were fixed and dropped on Fisher-brand™ Superfrost Plus Microscope Slides (FIS#12–550-15; Fisher Scientific). Metaphase spreads were dehydrated and subjected to T-FISH as described above.

### G_2_/M phase telomere synthesis assay with EdU-Click reaction

Cells on coverslips were arrested with 18 μM RO-3306 (SML0569, Sigma-Aldrich) for 16 h, followed by incubation with 20 μM EdU (5-ethynyl 2′-deoxyuridine) for 1 h. Cells were fixed then subjected to EdU-Click reaction using sulfo-Cyanine5 azide (A3330, Lumiprobe) in staining solution for 30 min. The coverslips were washed with phosphate-buffered saline (PBS) and then subjected to T-FISH as described above.

### Microscopy and time-lapse imaging

Fluorescence microscopy images were acquired using a CoolSNAP HQ^2^ cooled CCD camera on a DeltaVision Spectris Restoration microscope built around an Olympus IX70 stand with a ×60 numerical aperture 1.42 lens (GE Healthcare Life Sciences). The images were deconvoluted using the iterative algorithm in softWoRx software (GE Healthcare Life Sciences). Time-lapse images were acquired using a Nikon A1 laser scanning confocal microscope equipped with a heated stage at 37°C. Multicolor images were taken with 6 s time intervals for mCherry (561 nm) channels.

### Chromatin immunoprecipitation (ChIP)

Cells were cross-linked with 1% formaldehyde for 30 min, then quenched with glycine. Cell lysates were sonicated, pre-cleared and then incubated with the indicated antibodies at 4°C overnight. The next day, beads were added, incubated for 2 h then washed four times. Chromatin was eluted, reverse-cross-linked at 65°C overnight and blotted to a membrane. Results from hybridization with telomere or Alu probes were obtained by exposure to Typhoon™ FLA 7000 (GE Healthcare Life Sciences).

### Biotinylated oligonucleotide pull-down assay

Biotinylated DNA and RNA oligonucleotides (TTAGGG)_8_, (UUAGGG)_8_ and (CCCUAA)_8_ were purchased from Bioneer. Oligonucleotides were hybridized by mixing with either (CCCTAA)_8_ or (CCCTAA)_3_ and heated for 10 min at 95°C, then gradually cooled down to room temperature. Probes were bound to streptavidin–agarose beads (69203–3CN, Merck Millipore), then incubated with cell lysates at 4°C overnight. The protein–nucleic acid complexes were analyzed by immunoblot.

### Analysis of APB in paraffin-embedded human breast cancer specimens

Breast cancer samples from 38 patients were obtained as paraffin-embedded tissue sections from the Seoul National University Hospital. Samples were collected according to the research protocol approved by the Institutional Review Board of Seoul National University Hospital (H-1103-159-357; H-1707-174-874) and Seoul National University (E2208/004-003). Of the 38 patients, 19 had hereditary breast cancer with a deleterious germline mutation of *BRCA2*, and the other 19 had sporadic breast cancer without a *BRCA2* mutation. *BRCA2* mutations were confirmed with sequencing. Immuno-FISH analysis for APB detection was carried out as described (26). Samples were stained using a TelC-Cy3 PNA (peptide nucleic acid) probe, PML antibody and 4′,6-diamidino-2-phenylindole (DAPI). TFL-Telo software was used to analyze telomere length.

## Results

### Phase separation plays a critical role in facilitating break-induced replication (BIR)-mediated telomere synthesis in ALT-like cells

Depletion of Brca2 in telomerase-negative cells has been shown to induce BIR at telomeres and to initiate ALT-like activity (6,7). One of the hallmarks observed in these Brca2 deficiency-induced ALT-like cells is the appearance of PML bodies at telomeres, known as ALT-associated promyelocytic leukemia bodies (APBs) (6). To investigate the trigger responsible for APB assembly upon Brca2 depletion, we utilized MEFs where Brca2 could be conditionally deleted. *Brca2^F11/F11^*;*ER-Cre* mice were bred with *mTR^+/–^* mice to create *Brca2^F11/F11^*;*ER-Cre; mTR^+/+^* (*BI*, Brca2-inducible) or *Brca2^F11/F11^*;*ER-Cre; mTR^−/−^* (*TBI*, Telomerase-null and Brca2-inducible) offspring. BI and TBI MEFs were then isolated and immortalized by expressing the large T antigen of SV40 (6,9). In these BI and TBI fibroblasts, *Cre* expression was induced (6).

**Figure 1.**
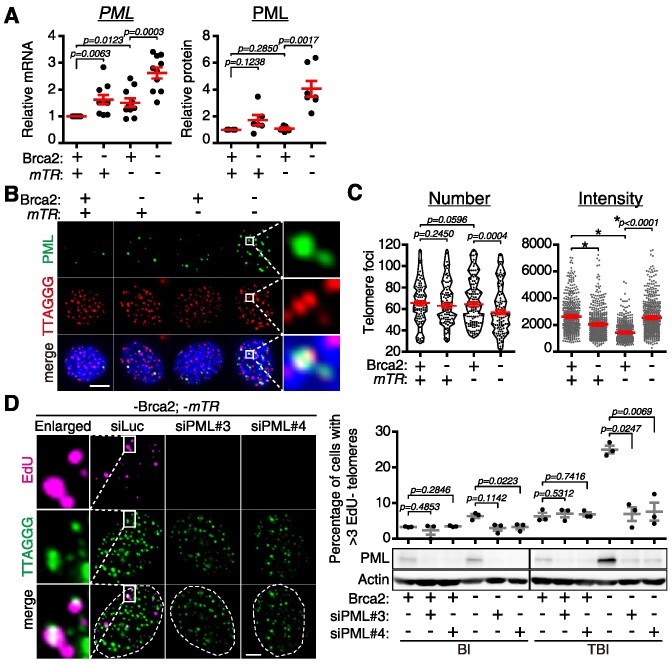
Assembly of PML bodies is essential for telomere synthesis in BRCA2-depleted ALT-like cells.(**A**) Comparison of *PML* mRNA (left) and protein (right) levels in telomerase-positive (+*mTR*) and telomerase-negative (–*mTR*) MEFs, in the presence or absence of *Brca2*. Fibroblasts from BI or telomerase-null TBI mice (6) were utilized to conditionally deplete *Brca2*. Tamoxifen (4-OHT) treatment selectively depletes the *Brca2^F11/F11^* allele. The dot graph is the result of MEFs from three different mice each to avoid individual difference. RT–qPCR was conducted in two technical replicates (left) and western blot was performed in one sample from three different animals each (right, Supplementary Figure S1A). (**B**) Representative images of APB in BI and TBI fibroblasts, in the presence or absence of Brca2. PML, green; telomere, red. Enlarged images of APB from the white square are shown on the right. Scale bar, 5 μm. (**C**) The number and intensity of telomere foci from (B) were scored. Graphs are from three independent experiments. More than 100 cells were scored to assess telomere number in each experiment. More than 500 foci from 50 different cells each were scored per experiment to assess the intensity. (**D**) Fibroblasts were transfected with two different siRNAs (*siPML*#3 and *siPML*#4) targeting *PML*, with or without *Brca2*, then subjected to analysis of telomere synthesis in G_2_ phase. (Left) Images representing the degree of EdU incorporation at telomeres in *Brca2* and telomerase double knockout TBI fibroblasts. Fibroblasts were treated with 18 μM CDK1 inhibitor RO-3306 to arrest cells in G_2_ phase, then subjected to assay of EdU incorporation at telomeres as described in the Materials and Methods. Enlarged images of EdU-positive telomeres in the control are shown on the left. Scale bar, 5 μm. (Right) Cells with more than three EdU-positive telomeres were scored. The graph is the result of three independent experiments (each dot corresponds to a single experiment). More than 100 BI (+*mTR*) and >200 TBI (–*mTR*) fibroblasts were scored in each experiment. Western blot analysis to evaluate the efficiency of PML knockdown after siRNA transfection is shown at the bottom. *P< 0.0001, Student's *t*-test (mean ± SEM).

First, we investigated whether Brca2 depletion affects PML levels. RT–qPCR analysis from MEFs generated from three different animals revealed that *PML* RNA increased 2-fold upon Brca2 depletion (Figure 1A, –Brca2; +*mTR*). Loss of telomerase exhibited a similar effect on RNA levels (Figure 1A, +Brca2; –*mTR*). Notably, *PML* expression was further increased 3-fold when both Brca2 and telomerase were depleted (Figure 1A, left, –Brca2; –*mTR*). Western blot analysis confirmed that Brca2 depletion resulted in an increase in PML levels (Figure 1A, right; Supplementary Figure S1A).

Immunofluorescence analysis revealed that PML co-localizes with telomeres only in Brca2-deficient and telomerase-null TBI fibroblasts (Figure 1B). Notably, two or more telomeres co-localized with PML (Figure 1B, enlarged). The number of telomeres decreased, and the intensity, reflecting the size of telomeres, increased in Brca2-deficient and telomerase-null TBI cells (Figure 1C), confirming that telomeres cluster at PML bodies in Brca2-deficient ALT-like MEFs (6). Depletion of BRCA2 with siRNA resulted in an increase of PML and APB, which was restored to the normal level upon ectopic expression of *BRCA2* using an siRNA-resistant *BRCA2-GFP-*expressing construct in HeLa LT *TERC* KO cells (Supplementary Figure S1B), confirming that BRCA2 abrogation indeed leads to the increase of PML in mouse and human cells.

We proceeded to investigate whether PML is critical in Brca2-deficient ALT-like cells. To this end, TBI fibroblasts, characterized by BIR and ALT-like activity (6), were depleted of PML, and telomere synthesis activity was assessed. Given that BRCA2 disruption induces telomere damage during replication in S phase (9), G_2_ or mitotic telomere synthesis is likely to be responsible for ALT upon BRCA2 loss (6). Therefore, we treated the Brca2-depleted TBI fibroblasts with the CDK1 inhibitor RO-3306 to arrest the cells at G_2_ phase. Subsequently, these cells were pulsed with EdU, followed by the measurement of telomere synthesis activity using EdU-Click assay coupled with T-FISH.

PML depletion with siRNA resulted in the abrogation of EdU incorporation at telomeres in Brca2-depleted TBI fibroblasts, which was markedly elevated upon disruption of Brca2 (Figure 1D, TBI; –Brca2; +siPML). It is noteworthy that the effect of Brca2 abrogation and PML depletion on telomere synthesis was marginal in BI fibroblasts (Figure 1D, right, BI), indicating that this EdU-Click G_2_ telomere synthesis assay monitors ALT-like activity. Taken together, the abrogation of Brca2 increases PML, which is essential for APB assembly. Furthermore, APB is critically required for ALT-like telomere synthesis.

The APB has been shown to exhibit LLPS properties (2,6); it has been suggested that promoting telomere clustering by inducing liquid condensate and enriching DNA repair factors in APBs are uncoupled events (14). As BRCA2 depletion led to an increase in PML and induced APB formation, we investigated whether BRCA2 abrogation is associated with the initiation of phase separation. Additionally, we aimed to determine whether the phase separation property, manifested by APB assembly, is essential in ALT activity or if phase separation is merely the result of proteins aggregating at the telomeres. To address these questions, we devised an experimental system capable of artificially inducing phase separation at telomeres and substituting for PML.

**Figure 2.**
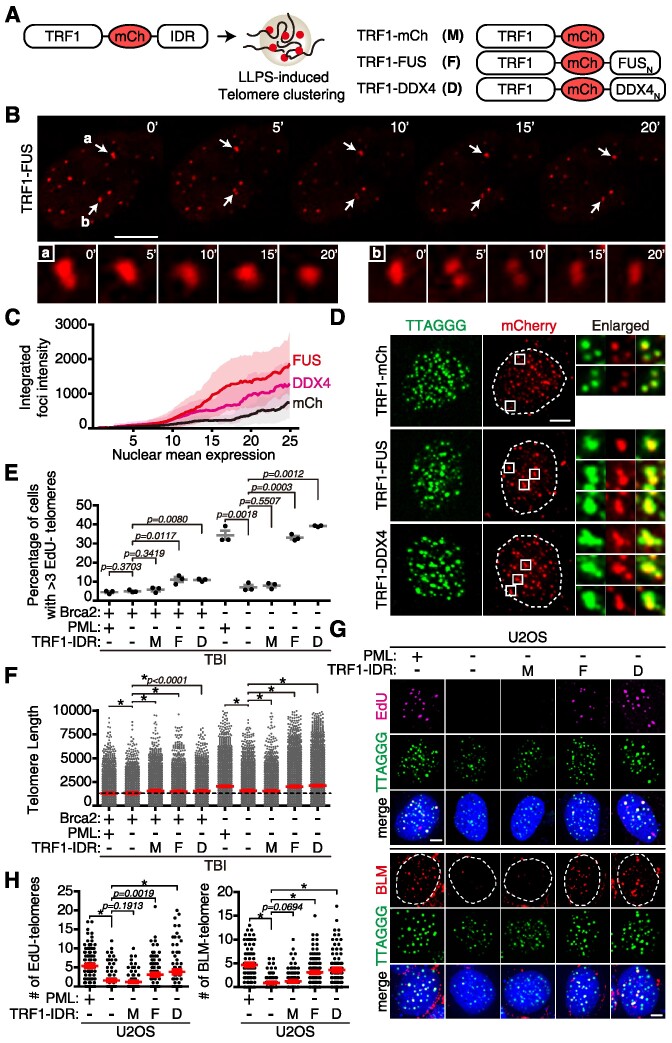
LLPS plays a pivotal role in telomere synthesis of ALT-like MEFs.(**A**) Illustration of *TRF1-IDR*-expressing constructs. The IDR domains from two different IDR-containing proteins, the N-terminus of FUS (*FUS_N_*) and the N-terminus of DDX4 (*DDX4_N_*), respectively, were fused to the *TRF1* expression construct. mCherry (mCh) fluorescence protein-expressing cDNA was inserted after *TRF1*. (**B**) Representative time-lapse captured images of TRF1–FUS movements in wild-type fibroblasts. Telomere foci forming into droplets are marked with arrows (a, b) and the enlarged images are shown below. Scale bar, 10 μm. (**C**) Integrated fluorescence intensity of *TRF1-IDR* clusters in the nucleus per cell are shown as arbitrary units on the *y*-axis. More than 400 cells were scored. x/y, integrated intensity of TRF1 foci/mean intensity of mCherry in the nucleus. Black, TRF1–mCh; red, TRF1–FUS; pink, TRF1–DDX4. (**D**) Fixed images of telomere clustering after transduction of TRF1-IDR-expressing lentivirus to wild-type fibroblasts. Cells were fixed and subjected to T-FISH coupled with immunofluorescence with anti-mCherry antibody. Note the telomere clusters in TRF1–FUS- or TRF1–DDX4-expressing fibroblasts in enlarged images. The number of telomere foci decreased upon clustering in *TRF1–FUS* (average foci number, 76.7) or *TRF1–DDX4* (77.2) expression, compared with control (TRF1–mCherry, 82.3). Telomere, green; mCherry, red. Scale bar, 5 μm. (**E**and **F**) TBI fibroblasts were transfected with *siPML* in the presence (+) or absence (–) of Brca2, followed by transduction with the indicated lentivirus: M, TRF1–mCh; F, TRF1–FUS; D, TRF1–DDX4. (**E**) Percentage of cells with >3 EdU-positive telomeres. The graph is the result of three independent experiments. More than 120 cells were scored in each set. (**F**) Comparison of telomere length. Telomere lengths were measured using T-FISH from metaphase chromosomes and are shown as arbitrary units of fluorescent intensity. More than 5000 telomere foci from > 50 metaphase spreads were scored in each experimental set. (**G** and **H**) U2OS cells were transfected with *siLuc* (control, +) or *siPML* (–), followed by transduction with the indicated lentivirus: M; F; D. (**G**) (Top) Representative images of EdU (pink) incorporation at telomeres (green) in G_2_ phase of each experimental set are shown. (Bottom) Representative images of BLM (red) at telomeres (green) are shown. Scale bar, 5 μm. (**H**) (Left) EdU-positive telomeres in U2OS cells. More than 100 cells were counted in each experimental set. (Right) BLM-positive telomeres in U2OS cells. More than 120 cells were scored in each set. *P< 0.0001, Student's *t*-test (mean ± SEM).

Previously, it has been demonstrated that intrinsically disordered regions (IDRs) contribute to promiscuous interactions, synergizing with specific protein–protein interactions and RNA–protein interactions, thereby providing a basis for LLPS (27,28). Specifically, IDR-harboring FUS (29) and DDX4 (30) were shown to induce LLPS. Taking this information into account, we generated lentiviral constructs that express IDRs from FUS or DDX4, fused to the telomere repeat-binding protein, TRF1. To ensure that secondary functions in these proteins do not interfere with ALT, only the N-terminus of FUS (FUS_N_) or DDX4 (DDX4_N_) encompassing the IDR was fused to TRF1. Additionally, the *mCherry*-expressing gene was fused between *TRF1* and *IDR* to enable visualization by microscopy (Figure 2A). Subsequently, these lentiviruses were introduced into cells in the presence or absence of PML, and the ability to form liquid condensates and G_2_ telomere synthesis activity were then analyzed.

Ectopic expression of TRF1-IDR resulted in liquid condensation at telomeres (Figure 2B), leading to telomere clustering analogous to the telomere clustering observed in the APB (2,6). During transcriptional control, liquid condensation demonstrates dynamic and selective interactions between domains (31). We investigated whether TRF1-IDR-induced liquid condensates display similar dynamic interactions using a fluorescence recovery after photobleaching (FRAP) assay. When the clustered telomeres were photobleached, they exhibited rapid recovery, corroborating that they display characteristics of liquid condensates (Supplementary Figure S2A).

The degree of telomere clustering was compared by scoring the integrated intensity of each telomere focus after ectopic expression of different *TRF1-IDR* constructs. The resulting data were then normalized to the mean intensity of nuclear mCherry fluorescence. The results showed that *TRF1–FUS* expression exhibited the highest fluorescence intensity in foci, followed by *TRF1–DDX4*. Control *TRF1–mCh* expression displayed the weakest fluorescence (Figure 2C). The expression level of *TRF1-IDRs* was similar, hence they did not affect the result (Supplementary Figure S2B). To corroborate that TRF1-IDR-induced clustering occurs at telomeres, cells were fixed and subjected to immunofluorescence with anti-mCherry antibody, coupled with T-FISH. It was observed that TRF1-IDR-induced clustering reflected telomere clustering as mCherry and T-FISH signals co-localized, forming clustered bodies (Figure 2D, enlarged). These results indicate that TRF1-IDR-induced artificial phase separation drives telomere clustering.

Using this TRF1-IDR system, we next investigated whether artificially engineered phase separation can substitute for PML-induced APB in telomere synthesis. TBI fibroblasts were depleted of PML and transduced with *TRF1-IDR-*expressing lentivirus in the presence or absence of Brca2. Abrogation of Brca2 in telomerase-null TBI fibroblasts caused a marked increase in G_2_ telomere synthesis (Figure 2E, –Brca2), accompanied by elongation of telomere length (Figure 2F, –Brca2). Depleting PML reduced G_2_ telomere synthesis (Figure 2E, –Brca2; –PML) and shortened telomere length (Figure 2F, –Brca2; –PML). Ectopic expression of *TRF1-IDR*, *-FUS*(*F*) or *-DDX4*(*D*) fully rescued the G_2_ telomere synthesis activity of PML-deficient cells to a level comparable with PML-proficient cells (Figure 2E, TBI, F and D). Concordantly, PML depletion-induced telomere shortening was recovered by the expression of TRF1–FUS or TRF1–DDX4 (Figure 2F, TBI, F and D). These effects were not observed in telomerase-proficient BI cells (Supplementary Figure S2F, G).

Introduction of NLS-IDR, where TRF1 was replaced with two nuclear localization signal (NLS) sequences adopted from SV40 (SV40-NLS) (Supplementary Figure S2C), generated large condensates in the nucleus (Supplementary Figure S2C). However, inducing phase separation in non-telomeric regions did not reactivate telomere synthesis activity that was lost from PML abrogation (Supplementary Figure S2D, E).

We next investigated whether human ALT cancer cell lines behave similarly. U2OS is a well-defined ALT cancer cell line which exploits BIR for telomere maintenance (1). In BRCA2-proficient U2OS cells, PML depletion resulted in a significant decrease in telomere synthesis (Figure 2G). Similar to ALT-like mouse TBI fibroblasts, the introduction of TRF1-IDR (Supplementary Figure S2H) recovered G_2_ telomere synthesis activity (Figure 2G, top).

BLM helicase localizes to APB in human ALT cell lines and is essential for BIR (15). When PML was depleted from U2OS cells, BLM-positive telomeres were markedly reduced (Figure 2G, bottom). Notably, BLM localization to telomeres was restored to a level comparable with the control upon ectopic expression of *TRF1-IDR* in PML-depleted cells (Figure 2G, F and D), supporting that induction of phase separation is the key to assemble BIR machinery for ALT activity in human cancer cells. As U2OS cells are proficient with BRCA2, the data imply that the pivotal role of phase separation in telomere synthesis is not restricted to BRCA2-deficient cells but is extended to BRCA2-proficient ALT cancer cells.

### BRCA2 deficiency results in telomere G4 stabilization and leads to phase separation

Despite the successful induction of artificial telomere LLPS, the introduction of TRF1-IDR did not initiate telomere synthesis when Brca2 is present (Figure 2E, F, +Brca2). This indicates that in addition to the assembly of telomeric LLPS, Brca2 depletion may be critical for the recruitment of an appropriate recombination machinery to the telomere phase separation. Previously, we reported that the dynamic interaction of BRCA2 with telomere G4 guarantees telomere replication homeostasis: BRCA2 interacts with G3 intermediates, which form during the interconversion between two different conformations of G4 (9). In effect, BRCA2 abrogation results in stabilization of telomere G4 due to the loss of dynamic interconversion. We asked whether abnormally stabilized telomeric G4 is responsible for the phase separation at telomeres.

**Figure 3.**
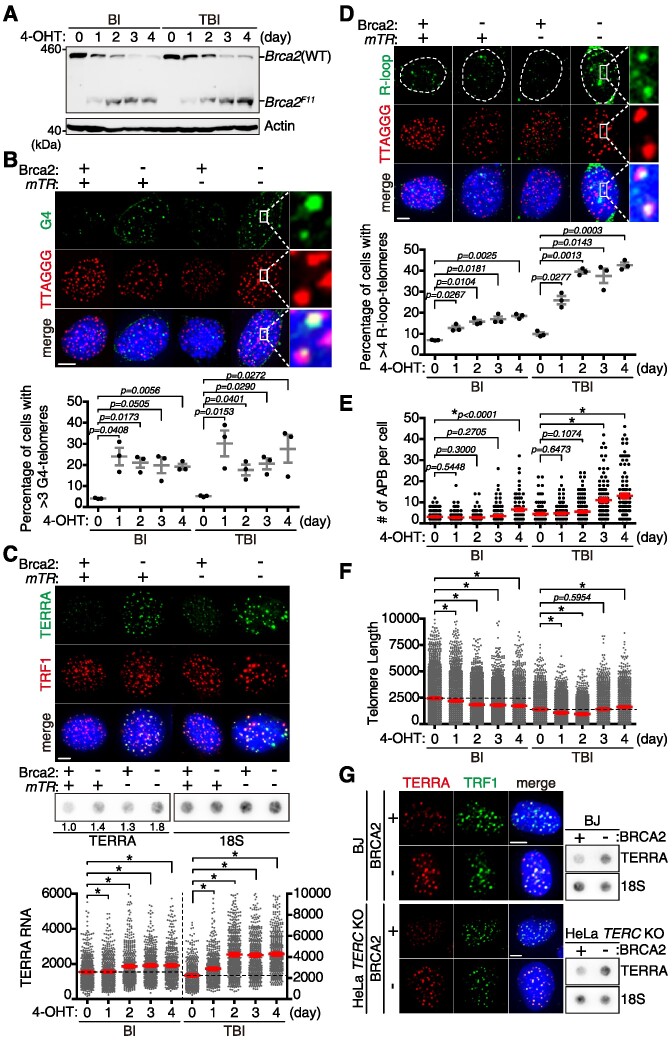
Stabilization of telomere G4 is associated with the increase of TERRA-R-loops, which leads to the assembly of telomeric PML bodies and telomere synthesis. (**A–F**) Experiments were performed in BI and TBI fibroblasts. For conditional depletion of Brca2, fibroblasts were treated with tamoxifen (4-OHT) and the indicated characteristics were analyzed everyday (B-F). (**A**) Western blot analysis of *Brca2* deletion and the creation of the *Brca2^F11^* allele upon 4-OHT treatment. (**B**) Assessing G4-positive telomeres. (Top) Representative images of G4 (green) and telomere (red) co-localization after 4 days of tamoxifen treatment. Fibroblasts were fixed and subjected to immunofluorescence with BG4 (anti-DNA G4 monoclonal antibody; green), followed by T-FISH with the TelC-PNA probe (red). Enlarged images of the white squares are shown on the right. (Bottom) Percentage of cells with more than three G4-positive telomeres is shown at the bottom. More than 100 cells each were scored in all sets. The graph is the result of three independent experiments. Scale bar, 5 μm. (**C**) Assessing TERRA RNA levels with TERRA-FISH and RNA dot-blot. (Top) TERRA RNA-FISH coupled with immunofluorescence assay. Immunofluorescence with anti-TRF1 antibody (red) was determined after 4 days of tamoxifen treatment, followed by TERRA RNA detection with TelC-PNA hybridization at 37°C (green). Scale bar, 5 μm. (Middle) Total RNA was extracted from fibroblasts after 4 days of Brca2 depletion, then dot-blotted. Radiolabeled *TERRA* and 18S rRNA probes were employed for hybridization. (Bottom) Quantification of TERRA RNA measured by RNA-FISH, shown as arbitrary units of fluorescent intensity. More than 500 TERRA foci from 40 cells each were scored. (**D**) Assessing R-loops at telomeres via immunofluorescence with S9.6 (anti-DNA:RNA hybrid antibody, green), coupled with T-FISH (red). Analysis was performed 4 days post-Brca2 depletion. Enlarged images from the white square are shown on the right. The graph represents the percentage of cells with >4 R-loop-positive telomeres. More than 120 cells each were scored. (**E**) Quantification of APBs per cell. More than 100 cells each were scored. (**F**) Telomere lengths were measured using T-FISH from metaphase spreads and are shown as arbitrary units of fluorescent intensity. More than 3000 telomere foci from > 50 metaphase chromosome spreads were measured. (**G**) Abundance of TERRA RNA levels in BJ fibroblasts and HeLa LT *TERC* KO cell lines with or without BRCA2. Both HeLa LT *TERC* KO and BJ cells were transfected with *siLuc* (+) or *siBRCA2* (–) for 3 days prior to fixation. Representative images of TRF1 (green) and TERRA (red). Scale bar, 2.5 μm. (Right) RNA dot-blot hybridized with TERRA or 18S rRNA probes. All the graphs are from three independent experiments. *P< 0.0001, Student's *t*-test (mean ± SEM).

Using G4-specific monoclonal antibody BG4 in immunofluorescence (Supplementary Figure S3A), we assessed the effect of Brca2 loss on telomeric G4. Brca2 depletion (Figure 3A, +4-OHT) led to a marked increase in telomeric G4 in both BI and TBI fibroblasts on day 1 post-Brca2 depletion (Figure 3B). The effect of Brca2 depletion on G4 increase was more severe in telomerase-negative TBI cells compared with the telomerase-positive BI cells on day 1 post-Brca2 depletion (Figure 3B, graph).

Losing the dynamicity of interconversion between two different G4 conformations will stabilize G4, resulting in stalling of replication forks. The stalled replication forks, if unresolved, lead to transcription–replication conflict (TRC), leading to an increase in unscheduled telomere RNA (TERRA) transcription, subsequently generating the DNA:RNA hybrid, the R-loop (8). Therefore, we analyzed if the level of TERRA and R-loop increases after telomere G4 stabilization. The abundance of TERRA, detected by RNA-FISH (Figure 3C, top; Supplementary Figure S3B) and RNA dot-blot with or without Brca2, was assessed (Figure 3C, middle; Supplementary Figure S3C). TERRA mostly co-localized with TRF1 (Figure 3C), and the abundance, measured by RNA dot-blot, increased when Brca2 was abrogated (Figure 3C, middle, –Brca2, ∼1.4-fold) or telomerase was absent (Figure 3C, middle, –*mTR*, ∼1.3-fold). Notably, Brca2-deficient and telomerase-null cells exhibited a marked increase in TERRA (Figure 3C, middle, –Brca2; –*mTR*, ∼1.8-fold).

Using RNA dot-blot, TERRA half-life was measured in the presence of the transcription inhibitor actinomycin D. It showed that TERRA half-life was >6 h in wild-type control, which extended to ∼8 h in Brca2-deficient or telomerase-null cells (Supplementary Figure S3C).

When the TERRA level was measured daily using immunofluorescence coupled with RNA-FISH, it showed that TERRA increased from day 1 post-Brca2 depletion in TBI fibroblasts (+4-OHT, Day 1, TBI), whereas it was delayed by 1 day in the presence of telomerase (+4-OHT, Day 2, BI). From day 2 post-Brca2 depletion (+4-OHT), Brca2-depleted and telomerase-deficient double null TBI fibroblasts exhibited a further increase of TERRA levels (Figure 3C, bottom, +4-OHT, TBI), compared with telomerase-positive BI fibroblasts (Figure 3C, BI).

Congruent with previous reports (32,33), immunofluorescence using the R-loop-specific monoclonal antibody S9.6 confirmed the increase of the R-loop upon Brca2 abrogation (Figure 3D). Telomeric R-loops were particularly elevated in telomerase-null TBI fibroblasts from day 1 post-Brca2 depletion, which further increased from day 2 post-depletion (Figure 3D, TBI).

The number of APBs significantly increased from day 3 post-Brca2 depletion in TBI fibroblasts (Figure 3E, TBI). However, in the presence of telomerase, the effect of Brca2 depletion was marginal (Figure 3E), albeit a slight increase of APB upon Brca2 depletion in telomerase-positive fibroblasts on day 4 post-Brca2 depletion (Figure 3E, +4-OHT, Day 4, BI). Concordant with the increase of APB, telomeres that shortened due to telomerase deficiency (–*mTR*) were elongated from day 3 post-Brca2 depletion in TBI fibroblasts (Figure 3F, +4-OHT, Day 3, TBI); elongation of telomeres was not observed but was maintained from day 2 post-Brca2 depletion in a telomerase-positive background (Figure 3F, +4-OHT, BI). Collectively, the absence of Brca2 led to the assembly of APB, the telomere LLPS and telomere synthesis in a sequential manner: an increase of telomeric G4 due to abnormal stabilization; transcription of TERRA; increase of TERRA-R-loops, particularly in a telomerase-deficient background; phase-separated APB assembly; and telomere elongation. That a telomerase-deficient background exhibited a profound effect upon Brca2 depletion in mice suggests that absence of telomerase activity plays a pivotal role in triggering ALT or ALT-like activity.

It is noteworthy that more R-loops are generated at short telomeres (34,35). As mouse telomeres are significantly longer than those of human, we asked if the above effects still hold true in human cells. We measured and compared TERRA-R-loop levels in HeLa cells devoid of *TERC* (HeLa LT *TERC* KO) (19) and BJ fibroblasts devoid of telomerase in the presence or absence of BRCA2. The level of TERRA, measured by both immunofluorescence coupled with RNA-FISH and dot-blot, was markedly elevated upon BRCA2 depletion in human cells, confirming the data from mouse fibroblasts (Figure 3G).

### Telomeric R-loops are essential in phase separation

**Figure 4.**
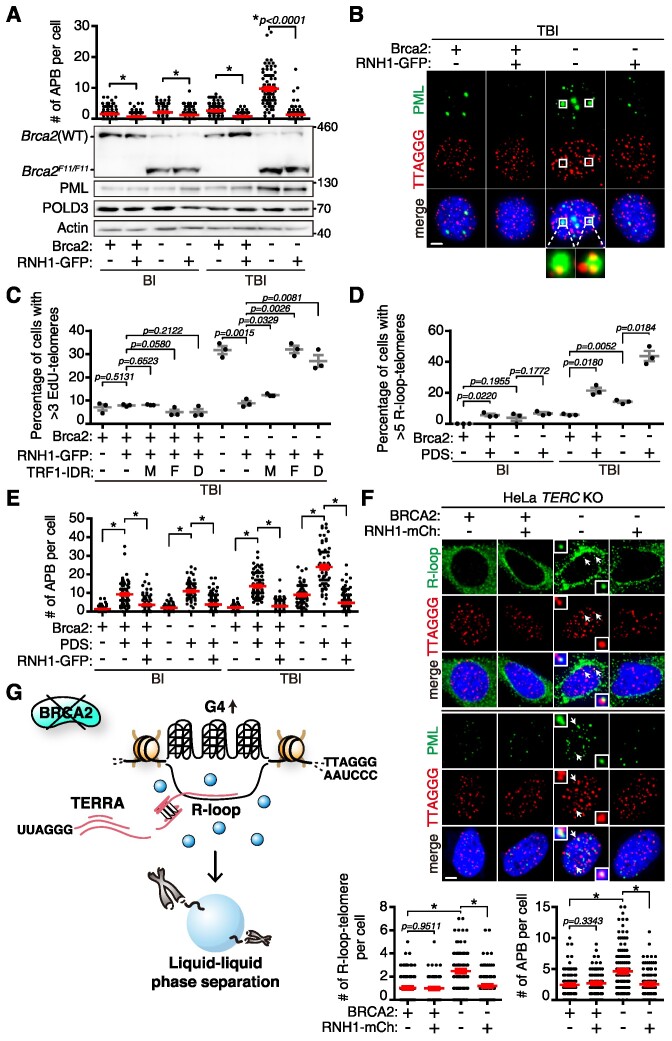
Telomeric R-loops drive LLPS formation. (**A–E**) Telomerase-positive BI and telomerase-negative TBI fibroblasts were transduced with lentivirus encoding *RNase H1-GFP* (*RNH1–GFP*). Fibroblasts expressing ectopic *RNH1* were selected with puromycin. (**A**) (Top) Number of APBs per cell. More than 100 cells each were scored. (Bottom) Western blot analysis to assess deletion and creation of the *Brca2^F11^* allele after 4-OHT treatment. The effect of Brca2 depletion and/or *RNH1–GFP* overexpression was assessed. The same blot was re-probed with anti-PML and anti-POLD3 antibodies. (**B**) Representative fluorescent images showing APB in TBI fibroblasts with or without *RNH1–GFP* expression in the presence (+) or absence (–) of Brca2. PML, anti-PML immunofluorescence (green); telomere, T-FISH (red). Enlarged images of APBs showing the telomere clustering from the white square are shown below. Scale bar, 5 μm. (**C**) Effect of R-loop formation and LLPS in G_2_ telomere synthesis. TBI fibroblasts expressing *RNH1–GFP* were transduced with lentivirus expressing *TRF1–mCh* (M), *–FUS* (F) and *–DDX4* (D), respectively, then subjected to G_2_ telomere synthesis assay. The graph showing the percentage of TBI cells with >3 EdU-positive telomeres is from three independent experiments. More than 100 cells each were scored. (**D**) Effect of G4 stabilization in telomere R-loop accumulation. BI or TBI fibroblasts, treated with 4-OHT (–) or left untreated (+) to deplete *Brca2*, were exposed to 5 μM G4 stabilizer pyridostatin (PDS) for 2 h. Cells untreated with PDS were included as control. The percentage of cells with >5 R-loop-positive telomeres is marked. More than 120 cells each were scored. (**E**) Effect of PDS and/or RNH1 on APB formation. More than 100 cells each were counted. (**F**) HeLa LT *TERC* KO cells were transfected with *siLuc* (control) or *siBRCA2*, and simultaneously transfected with *mCherry*- or *RNH1–mCherry*-expressing constructs. (Top) Representative images of R-loop (green) and telomere (red) co-localization. Representative images of PML (green) and telomere (red) co-localization. Enlarged images from the white arrow are shown. Scale bar, 2.5 μm. (Bottom) Quantification of R-loop-positive telomeres and APBs from images on the top. More than 120 cells each were scored. (**G**) Model for how the absence of BRCA2 instigates ALT-like telomere synthesis. Brca2 depletion provokes stabilization of G4, and subsequently increases TERRA-R-loops. R-loops trigger LLPS at telomeres, which contain the protein complex required for BIR (6). All graphs are the result of three independent experiments. *P< 0.0001, Student's *t*-test (mean ± SEM).

The order of events above suggested the possibility that telomere R-loops may be essential in phase separation. We asked if APB assembly is affected when telomeric R-loops are eliminated. For this, we utilized the function of R-loop-specific nuclease, RNase H1, which specifically degrades RNA in DNA:RNA hybrids (36). The effect of GFP-tagged RNase H1 (*RNH1–GFP*) expression was corroborated in Brca2-depleted TBI fibroblasts. When *RNH1–GFP* was ectopically expressed in Brca2-depleted TBI cells (Supplementary Figure S3D and S4A), it led to a significant decline of phase-separated telomeric PML bodies (Figure 4A, B). The data indicate that the R-loop is involved in telomeric LLPS formation. By western blot, it was shown that *RNH1–GFP* expression did not interfere with POLD3 protein levels, essential in BIR (Figure 4A, POLD3 WB). Note that the up-regulated PML upon Brca2 depletion is not affected by *RNH1* expression, despite the decline of telomeric PML bodies (Figure 4A, PML WB).

Next, we asked whether telomere synthesis is affected when R-loops are disrupted. Thus we asked if artificially engineered phase separation at telomeres can functionally substitute the APB and rescue telomere synthesis activity in the absence of telomere R-loops. For this, *RNH1* was ectopically expressed in TBI fibroblasts in the presence (+) or absence (–) of Brca2. The cells were then transduced with *TRF1-IDR*-expressing lentivirus to engineer phase separation at telomeres. At 50 h post-transduction, G_2_/M-arrested cells were subjected to the EdU incorporation assay. The result showed that *RNH1–GFP* expression (Supplementary Figure S4A) markedly reduced telomere synthesis activity in Brca2-deficient TBI fibroblasts (Figure 4C, –Brca2; +RNH1–GFP). Of note, ectopic expression of *TRF1–FUS* (F) or *TRF1–DDX4* (D), but not control (M), rescued the G_2_ telomere synthesis that was lost upon *RNH1–GFP* expression in *Brca2*-depleted ALT cells (Figure 4C; Supplementary Figure S4B). In sharp contrast, expression of the non-telomeric nuclear IDR (*NLS-IDR*) did not have any effect on telomere synthesis (Supplementary Figure S4C) or telomere elongation (Supplementary Figure S4D). These results confirm that R-loops are required for phase separation instigation and that telomeric LLPS formation is essential for BIR-mediated telomere synthesis.

PDS is a G4-stabilizing ligand that induces DNA damage (37). It was shown that PDS-induced DNA damage sites are correlated with the spread of R-loops, suggesting that unscheduled G4 stabilization and increase of R-loops are linked in DNA damage and repair (38). We measured telomeric R-loops in cells with or without Brca2 and compared the data with cells treated with PDS. Depletion of Brca2 led to an increase of telomeric R-loops, as did PDS treatment. The effect of Brca2 depletion or PDS treatment on R-loop generation was more profound in the telomerase-null background (Figure 4D). Intriguingly, combining Brca2 loss and PDS treatment together further exacerbated the effect on R-loop generation (Figure 4D, TBI; –Brca2; +PDS).

The number of APBs increased upon PDS treatment or Brca2 abrogation in both telomerase-positive and negative cells; the effect was more profound in the telomerase-negative background (Figure 4E). Expression of *RNH1–GFP* abolished APBs both in PDS-induced and in Brca2 depletion-induced cells; *RNH1* expression was effective in abolishing APBs that were additively elevated upon loss of Brca2 and PDS treatment together in TBI fibroblasts (Figure 4E, –Brca2; +PDS; +RNH1–GFP). These results support the notion that stabilized G4 induces telomeric R-loop generation, and telomeric R-loops trigger the phase separation at telomeres.

We have assessed the effects of telomere breaks during replication when Brca2 is abrogated. However, abrogation of Brca2 leads to DNA damage throughout the genome. Furthermore, prolonged tamoxifen treatment can lead to DNA damage, which may affect the results. Therefore, we assessed the level of DNA damage by tamoxifen treatment. Immunofluorescence with γ-H2A.X showed that tamoxifen treatment leads to a slight increase of γ-H2A.X-positive foci, but the level was sustained for up to 4 days of treatment in control wild-type *Brca2^F11/F11^* MEFs lacking ER-Cre (Supplementary Figure S5A, +4-OHT). In comparison, Brca2 abrogation by introducing cells with lentiviral transduction-aided NLS-Cre resulted in a marked increase of γ-H2A.X-positive foci on day 4 post-transduction (Supplementary Figure S5A, NLS-Cre).

Telomere R-loops can form *in trans* in a RAD51-dependent manner as well (35), and may affect phase separation. However, the chance of such an effect of RAD51 is low as we were aware that RAD51 cannot locate in the damaged nuclear loci if BRCA2 is absent (39), which was recapitulated by the treatment with mitomycin C (MMC) (Supplementary Figure S5B). Consistent with this, we have also shown previously that RAD51’s function in restarting the stalled replication forks at the telomere is lost when BRCA2 is absent because RAD51 cannot locate to the stalled site (9). Nevertheless, the effect of RAD51 in telomere R-loop generation in BI and TBI fibroblasts was assessed in the presence or absence of Brca2. The result showed that depletion of Rad51 in Brca2-proficient cells resulted in a slight reduction of telomere R-loops; however, the effect of Rad51 was not observed in Brca2-deficient TBI fibroblasts (Supplementary Figure S5C), strongly supporting that telomere R-loop generation and phase separation in Brca2-deficient cells do not involve Rad51.

We next asked if human cells behave in a similar manner. HeLa LT *TERC* KO cell lines (19) and BJ fibroblasts that lack telomerase activity were depleted of *BRCA2* with siRNA (siBRCA2). *RNH1–GFP* was then ectopically expressed and the levels of telomeric R-loops were assessed. In parallel, APB formation was analyzed (Figure 4F; Supplementary Figure S6). Congruent with the results from mouse fibroblasts, depletion of BRCA2 in human cells devoid of telomerase increased the level of telomeric R-loops and telomeric PML bodies (Figure 4F, –BRCA2). Both R-loops and telomere PML bodies that increased upon BRCA2 depletion were reduced markedly when *RNH1* was expressed (Figure 4F; Supplementary Figure S6). These results corroborate that telomeric R-loops trigger phase separation in human ALT cells as well. We propose that abrogation of BRCA2 results in the loss of telomere G4 dynamicity, leading to the stabilization of G4. Abnormally stabilized telomere G4 causes TRC, resulting in the accumulation of TERRA-containing R-loops. Telomere R-loops trigger LLPS and BIR at telomeres. Collectively, loss of telomere G4 dynamicity is responsible for phase separation and BIR at telomeres (Figure 4G).

### Telomeres in ALT-like cells are marked with H3K27me3

The status of telomeric chromatin (euchromatic or heterochromatic) during ALT telomere synthesis remains controversial (8). Previously, we showed that BIR, which exploits a conservative replication mechanism, underlies ALT telomere synthesis upon BRCA2 loss. We also suggested that mitotic DNA synthesis, MiDAS (40,41), underlies this BRCA2 deficiency-induced ALT (6). These results suggest that telomere synthesis in BRCA2-deficient ALT-like cells may require the heterochromatin state.

**Figure 5.**
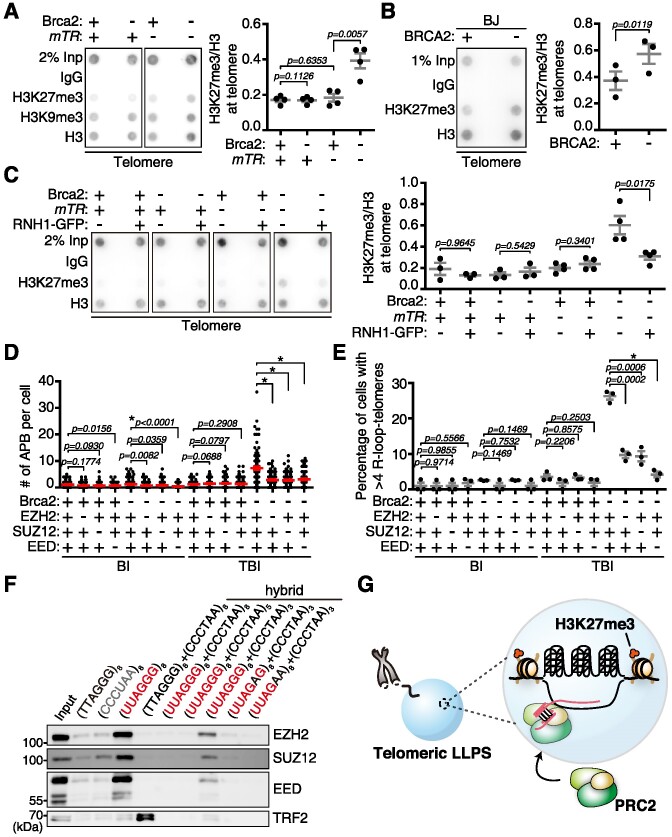
Telomeric R-loops are required for telomere H3K27 tri-methylation inside the liquid condensaste.(**A**) (Left) BI (+*mTR*) and TBI (–*mTR*) fibroblasts depleted of Brca2 (–) or left untouched (+) were subjected to ChIP against H3K27me3, H3K9me3 and histone H3, followed by hybridization with telomere probes. (Right) Relative H3K27me3 level at telomeres, normalized to histone H3. Four different BI and TBI MEFs, respectively, originating from different animals were employed in the analysis. (**B**) (Left) BJ fibroblasts transfected with *siLuc* (control) or *siBRCA2* were subjected to ChIP against H3K27me3 and histone H3. (Right) Abundance of H3K27me3 at telomeres, normalized to H3. The graph is the result of three independent experiments. (**C**) *RNH1*-expressing BI and TBI fibroblasts, in the presence or absence of Brca2, were subjected to ChIP against anti-H3K27me3 and -H3 antibodies, respectively, followed by hybridization with telomere probes. (Right) Level of H3K27me3 at telomeres, normalized to histone H3. The data represent three independent experiments. (**D** and **E**) BI and TBI fibroblasts were depleted of PRC2 core components EZH2, SUZ12 and EED proteins via siRNA transfection, in the presence (+) or absence (–) of Brca2. (**D**) Depleting the PRC2 complex abolished telomere LLPS. More than 100 cells each were counted. (**E**) Depletion of PRC2 markedly reduces telomeric R-loop generation. More than 100 cells each were counted. (**F**) Oligonucleotide pull-down assay to analyze the PRC2-recruiting region in telomere R-loops. Cell lysates were incubated with the indicated biotinylated oligonucleotides and subjected to western blot analysis with the indicated antibodies. RNA oligonucleotides (CCCUAA)_8_ and (UUAGGG)_8_ represent TERRA antisense and TERRA, respectively. The hybrid of (TTAGGG)_8_ + (CCCTAA)_8_ represents a double-stranded telomere, and (UUAGGG)_8_+ (CCCTAA)_8_ represents DNA:RNA hybrids (R-loops). (UUAGGG)_8_+ (CCCTAA)_5_ and (UUAGGG)_8_ + (CCCTAA)_3_ represent DNA:RNA hybrids with three and five repeats of exposed TERRA, respectively. (UUAGAG)_8_ + (CCCTAA)_3_ and (UUAGAA)_8_ + (CCCTAA)_3_ represent non-G4-forming sequences. (**G**) Illustration of the telomeric chromatin remodeling essential for ALT-like telomere synthesis inside the liquid condensate. TERRA RNA protruding from the R-loop recruits the PRC2 complex, which catalyzes H3K27me3 at telomeres. All results are from three independent experiments. *P< 0.0001, Student's *t*-test (mean ± SEM).

To uncover the molecular landscape of telomere chromatin in BRCA2 deficiency-induced ALT, we examined chromatin compaction by measuring the abundance of histone H3 tri-methylation at Lys9 (H3K9me3) and Lys27 (H3K27me3). BI and TBI fibroblasts with or without tamoxifen treatment were subjected to telomere ChIP with antibodies against H3K9me3 and H3K27me3. The ratios of H3K9me3 or H3K27me3 normalized to histone H3 were compared. H3K9me3 was detected in all telomeres (Supplementary Figure S7A, H3K9me3/H3). In comparison, the H3K27me3 level increased by ∼2-fold upon depletion of Brca2 in the telomerase-null background (Figure 5A, –Brca2; –*mTR*, H3K27me3/H3).

H3K27 tri-methylation is mediated by the Polycomb Repressive Complex (PRC2), comprised of three core proteins, EZH2, SUZ12 and EED (42,43). Telomere ChIP revealed that depletion of EZH2 resulted in a significant reduction of H3K27me3; H3K9me3 was decreased by ∼15% while histone H3 was unaffected (Supplementary Figure S7B). BRCA2 depletion led to an increase of telomeric H3K27me3 in human BJ cells as well (Figure 5B). Altogether, these results suggest that the H3K27me3-enriched heterochromatin state specifies telomeres upon BRCA2 loss.

Next, we asked whether the H3K27me3 mark is affected by R-loops. Ectopic expression of *RNH1–GFP* led to a significant decrease of H3K27me3-marked telomeres (Figure 5C, –Brca2; –*mTR*; +RNH1–GFP), indicating that an R-loop is required for H3K27me3 enrichment at telomeres. Then we asked if H3K27me3 marking is required for BIR-mediated telomere synthesis. Core components of PRC2 were depleted from immortalized fibroblasts and subjected to G_2_ telomere synthesis assay. Depletion of EZH2, SUZ12 or EED (Supplementary Figure S7C) significantly decreased the number of APBs (Figure 5D) and telomere synthesis (Supplementary Figure S7D) in Brca2-deficient TBI fibroblasts.

To confirm that H3K27 trimethylation is critical for BIR in ALT-like activity, EZH2 was inhibited using EPZ-6438, a SAM (*S*-adenosyl-methionine) competitor which specifically inhibits EZH2 activity (44) (Supplementary Figure S7E). The effect of EPZ-6438 on APB formation was marginal in telomerase-positive BI fibroblasts (Supplementary Figure S7F, BI; +/– Brca2). In contrast, EZH2 inhibition led to a decrease in APB number in Brca2-deficient and telomerase-null TBI fibroblasts (Supplementary Figure S7F, TBI; –Brca2). Note that PDS treatment of Brca2-deficient TBI cells markedly increased the number of APBs (Supplementary Figure S7F, TBI; –Brca2; –EZH2i; +PDS), which was decreased significantly upon EZH2 inhibition (TBI; –Brca2; +EZH2i; +PDS). PRC2 inhibition with the EZH2 inhibitor reduced APB in BRCA2-depleted human BJ fibroblasts as well (Supplementary Figure S7I). These results support that loss of telomere G4 dynamicity is responsible for telomeric LLPS formation. Enrichment of H3K27me3 at telomeres is associated with phase-separated APB.

In *Drosophila* PREs (Polycomb Response Elements), PRC2 contributes to R-loop formation through RNA:DNA exchange (45). Indeed, depletion of the PRC2 core complex resulted in marked reduction of R-loops at telomeres and TERRA transcripts (Figure 5E; Supplementary Figure S7H). However, it should be noted that induction of TERRA-R-loops was most significant upon treatment with the G4 stabilizer PDS in Brca2-deficient and telomerase-null cells, subsequently inducing APB assembly (Supplementary Figure S7F, G), suggesting that stabilized G4 induces TERRA-R-loop generation due to TRC. PRC2 may recognize TERRA (46) and be recruited to the telomere. Recruited PRC2 may in turn generate more R-loops and contribute to APB maintenance.

DNA polymerase δ subunit 3 (POLD3) (6) and BLM (15) are essential components of BIR. We asked whether POLD3 or BLM depletion affects LLPS formation (Supplementary Figure S7J). Abrogation of POLD3 or BLM disrupted APB formation, as well as telomere synthesis (Supplementary Figure S7J). BLM was shown to reside in phase-separated nuclear condensates (15). The result suggests that telomere LLPS is such a tight interaction network that perturbing any component of LLPS disrupts the droplet and may interfere with its function.

### TERRA-R-loops recruit PRC2 for H3K27me3 enrichment at telomeres in ALT-like cells

The above result suggested that TERRA-R-loops may recruit PRC2 and play a direct role in H3K27me3 marking of telomeres. Along this line, it was shown that the single-stranded non-coding RNA TERRA is responsible for the binding of PRC2 and mediates catalyzation of tri-methylation of Lys27 of histone H, and subsequent establishment of H3K9me3 for telomere heterochromatin formation (46). We asked whether TERRA-R-loops we capable of binding to PRC2, and if this interaction is essential in LLPS maintenance. DNA and/or RNA oligonucleotides representing various portions of the telomeric R-loop were biotinylated and subjected to pull-down experiments with EZH2, SUZ12 or EED immune complexes from Brca2-depleted TBI fibroblasts. EZH2, SUZ12 and EED were bound to TERRA RNA [Figure 5F, (UUAGGG)_8_], but not the complementary sequences [Figure 5F, (CCCUAA)_8_] nor the single-stranded telomere DNA [Figure 5F, (TTAGGG)_8_]. The telomeric DNA:RNA hybrid (UUAGGG)_8_ + (CCCTAA)_8_, which mimics a triplex R-loop without single-stranded TERRA, did not interact with any of the PRC2 proteins [Figure 5F, (UUAGGG)_8_ + (CCCTAA)_8_]. Of note, (UUAGGG)_8_ + (CCCTAA)_3_, which harbors single-stranded RNA protruding from the DNA:RNA hybrid, bound to all three PRC2 core components [Figure 5F, (UUAGGG)_8_ + (CCCTAA)_3_].

We next asked if a certain length of protruding TERRA was required for binding to PRC2. (UUAGGG)_8_ + (CCCTAA)_5_, which harbors three repeats of TERRA protruding from the DNA:RNA triplex, was not capable of binding to the PRC2 complex, whereas (UUAGGG)_8_ + (CCCTAA)_3_ that has five repeats of TERRA protruding from the DNA:RNA hybrid did (Figure 5F). Note that disruption of guanine repeats in TERRA abolished the binding [Figure 5F, (UUAGAG)_8_ + (CCCTAA)_3_; (UUAGAA)_8_ + (CCCTAA)_3_]. These data suggest that more than five repeats of single-stranded TERRA protruding from R-loops and guanine repeats are required to associate with PRC2. Taken together, a TERRA-R-loop recruits the PRC2 complex to telomere LLPS, and more than five repeats of TERRA protruding out from the DNA:RNA hybrid are required for the recruitment. PRC2 inside telomeric liquid condensates plays a pivotal role in the formation of telomere heterochromatin, which is required for BIR (Figure 5G).

### Clinical implications of targeting telomere G4 and LLPS in ALT-like cells

We investigated the clinical potential of targeting G4 dynamicity and the phase separation property. It has been shown that BRCA2-deficient cells are sensitive to treatment with the G4 stabilizer PDS (47). Our findings demonstrate that loss of BRCA2 results in abnormal stabilization of telomeric G4 due to a loss of dynamicity. Furthermore, treatment with PDS exacerbates the stabilization and accumulation of telomeric G4, leading to increased levels of R-loops and APBs. These results suggest that BRCA2-deficient cells, particularly those exhibiting ALT-like activity, may be susceptible to PDS treatment.

Previous studies have indicated that inhibition of EZH2 sensitizes BRCA2-deficient breast cancers to PARP inhibitors (48). Given that inhibition of EZH2 methyltransferase disrupts telomere LLPS maintenance to some extent in Brca2-deficient cells (Supplementary Figure S7E–G), and PDS treatment exacerbates G4 stabilization and R-loop generation, we sought to investigate whether co-treatment of PDS with an EZH2 inhibitor would selectively interfere with the growth and/or viability of Brca2-deficient and telomerase-null ALT-like cells.

**Figure 6.**
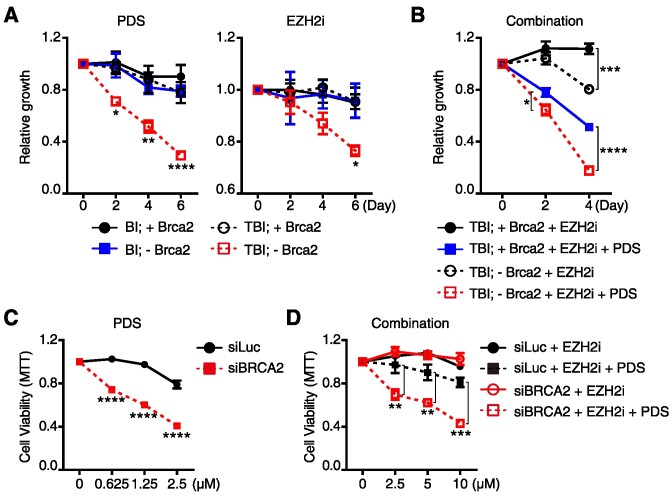
BRCA2-deficient ALT-like cells exhibit selective sensitivity towards G4 stabilizer and EZH2 inhibitor. (**A**) Relative growth of BI and TBI fibroblasts, with or without Brca2, upon PDS (left) or EPZ-6438 (right, EZH2i) treatment. The numbers of cells counted were normalized to non-treated cells to compare the rate of cell growth. Black line with circle, wild-type control; blue line with square, BI and –Brca2; black dotted line with open circle, TBI; red dotted line with open square, TBI and –Brca2. (**B**) Relative growth of TBI fibroblasts, with or without Brca2, upon combination of 2.5 μM PDS and 1 μM EZH2i. Cells were counted every 2 days and were normalized to non-treated cells to compare the growth rate. Black line with circle, TBI + EZH2i; blue line with square, TBI + EZH2i + PDS; black dotted line with open circle, TBI and –Brca2 + EZH2i; red dotted line with open square, TBI and –Brca2 + EZH2i + PDS. (**C** and **D**) Cell viability analysis of HeLa LT *TERC* KO cell lines with and without BRCA2 upon PDS and/or EPZ-6438 (EZH2i) treatment. HeLa LT *TERC* KO cells were transfected with *siLuc* (control) or *siBRCA2* for 24 h, then seeded in a 96-well plate and treated with the drug at the indicated concentrations. Three days later, cell viability was measured via MTT assay. (**C**) Graph showing the result of PDS treatment from 0 to 2.5 μM for 3 days. Black line with circle, siLuc; red dotted line with square, siBRCA2. (**D**) Graph showing the response to EZH2i and combined treatment with PDS and EZH2i in HeLa LT *TERC* KO cells, with or without BRCA2. Cells treated with 1 μM PDS were co-treated with 0–10 μM EPZ-6438. Black line with circle, control (siLuc + EZH2i); black dotted line with square, siLuc + EZH2i + PDS; red line with open circle, siBRCA2 + EZH2i; red dotted line with open square, siBRCA2 + EZH2i + PDS. The experiments were repeated three times independently. *P < 0.05, **P< 0.01, ***P< 0.001, ****P< 0.0001, Student's *t*-test (mean ± SEM).

BI and TBI fibroblasts with or without Brca2 were treated with 5 μM PDS or EPZ-6438, and the relative cell growth was assessed by scoring cell numbers, then the data were normalized to untreated (NT) cells. Values >1.0 on the *y*-axis indicate no response to the drug, and values <1.0 represent growth inhibition. Wild-type fibroblasts were unaffected by either PDS or EPZ-6438 (Figure 6A, black circle). The number of Brca2-deficient or telomerase-negative fibroblasts was slightly decreased by PDS treatment (Figure 6A). In comparison, Brca2- and telomerase-negative TBI cells exhibited significant growth suppression in response to PDS treatment (Figure 6A, PDS, red square). EPZ-6438 treatment did not show any growth inhibition in telomerase-null cells (Figure 6A, EZH2i, black open circle). However, EZH2 inhibition resulted in a moderate decrease of cell numbers in Brca2-deficient and telomerase-null double mutant fibroblasts (Figure 6A, EZH2i, red square).

The effects of combined treatment on TBI fibroblasts were assessed: growth rate was measured after treatment with 1 μM EPZ-6438 alone or upon co-treatment with 2.5 μM PDS. EZH2 inhibition moderately inhibited the growth of Brca2-deficient TBI cells (Figure 6B, black open circle), while Brca2-proficient telomerase-null cells were insensitive to EZH2 inhibition (Figure 6B, black circle). Combinatorial treatment of PDS with EPZ-6438 resulted in a significant inhibition of growth in Brca2-depleted TBI fibroblasts such that almost complete cell growth inhibition was observed 4 days post-treatment (Figure 6B, red square).

Next, we examined whether human cells also respond to this combination of inhibitors. HeLa cells were depleted of telomerase (HeLa LT *TERC* KO) then transfected with *siLuc* (control) or *siBRCA2*. The cells were then subjected to MTT [3,(4,5-dimethylthiazol-2-yl)-2,5-diphenyltetrazolium bromide] assay to measure cell viability, 3 days post-treatment with PDS, EPZ-6438, or EPZ-6438 and PDS together. PDS treatment suppressed cellular viability in BRCA2-depleted HeLa LT *TERC* KO cells, consistent with a previous report (47) (Figure 6C). Like mouse fibroblasts, EZH2 inhibition alone showed a minimal effect in human cells (Figure 6D). However, when EPZ-6438 and PDS were used for co-treatment, selective cytotoxicity in BRCA2-deficient ALT cells was observed (Figure 6D, red open square). These results suggest that perturbing the dynamicity of telomere G4 and LLPS maintenance interferes not only with BIR-mediated telomere synthesis but also with cell viability in Brca2-deficient ALT-like cells.

**Figure 7.**
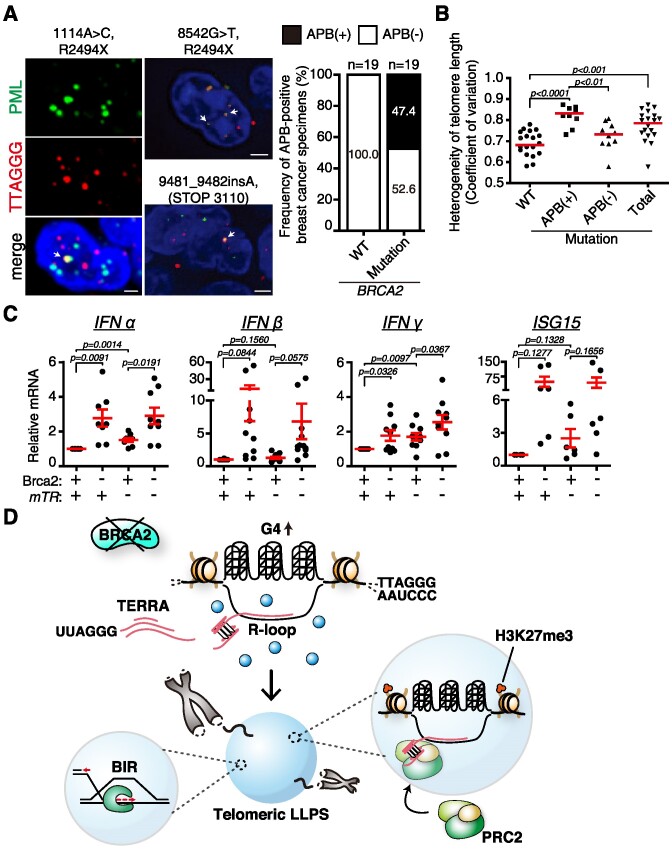
Clinical implication of the Brca2 deficiency and ALT development in breast cancer. (**A**) Approximately half of *BRCA2*-mutated human breast cancers display APBs. Representative fluorescence image of APB-positive human breast cancer tissues. Immunofluorescence with anti-PML antibody combined with T-FISH was performed in paraffin-embedded tissue sections. White arrow, APB. Bar graph, the frequency of APB-positive breast cancer specimens. Breast cancers of wild-type *BRCA2* (WT) and mutant *BRCA2* (Mutation) are compared. Nineteen samples each were employed. x/y, number of APB-positive sections/total number of samples. Scale bars, 2 μm. (**B**) Heterogeneity in telomere lengths (coefficient of variation) was calculated as the standard deviation of telomere length divided by the average telomere length. (**C**) Increase of inflammation-associated gene expression upon Brca2 depletion. Relative mRNA levels of *Interferon-α, -β, -γ* and *ISG15*, respectively, in telomerase-positive (+*mTR*) and telomerase-negative (–*mTR*) MEFs were assessed in the presence or absence of *Brca2*. The graph is the result of MEFs generated from three different animals. Two technical replicates were employed in every PCR. (**D**) Model of how BIR is triggered for telomere synthesis after BRCA2 abrogation. Disruption of BRCA2 stabilizes telomeric G4, which accumulates TERRA-containing R-loops at telomeres. Increased R-loops provoke telomere phase separation. At the same time, TERRA protruding from R-loops recruits PRC2 and marks telomeres with H3K27me3; BIR machineries are recruited due to the breakage and assembly of the liquid condensates facilitating telomere recombination. H3K27me3-marked telomeres are posed for conservative telomere synthesis, BIR. Student's *t*-test (mean ± SEM).

Lastly, we examined if *BRCA2* mutation is associated with human ALT cancers. Thirty-eight paraffin-embedded tissue sections of human breast cancer specimens were assessed for the presence of APBs by immunostaining with anti-PML antibody, coupled with T-FISH. The result showed that approximately half of BRCA2-mutated human cancers display APB positivity (47%, *n* = 19). None of the breast cancer specimens with wild-type *BRCA2* exhibited APBs (Figure 7A). Heterogeneity of telomere length, which is also a hallmark of ALT cancers (49), was elevated in APB-displaying *BRCA2*-mutated cancers (Figure 7B). Taken together, BRCA2 disruption can lead to ALT-type cancer. It should be noted that telomerase positivity and ALT activity can both exist in cancer cell lines and human tumors (49).

PML nuclear bodies have previously been implicated in the pathogenesis of human diseases. Increased PML expression has been observed during inflammation and in tumor tissue, suggesting potential roles of PML nuclear bodies in inflammation, cell growth control and other aspects of tumorigenesis (50). Given that depletion of Brca2 leads to an increase in PML RNA and protein levels, we investigated whether the elevation of PML affects the expression of pro-inflammatory cytokines. RT–qPCR analysis revealed that *interferon-α*(*IFN-α*), *IFN-β* and *IFN-γ* were all elevated upon Brca2 depletion, with the order of induction being *IFN-β* (∼30-fold) > *IFN-α* (∼4-fold) > *IFN-γ* (∼2-fold), with an additive effect in telomerase-null cells to ∼3-fold (Figure 7C). Telomerase deficiency alone resulted in only a slight increase compared with the control.

Recently, it was reported that IFN-β treatment restores replication fork stability and cell viability through interferon-stimulated gene 15 (ISG15) in BRCA2-defective cells, which is responsible for drug resistance in BRCA-defective triple-negative breast cancer (51). We assessed the level of ISG15 and found that, like IFN-β, the*ISG15* increase was significant (>75-fold) upon Brca2 abrogation, consistent with that report. These results suggest that BRCA2 deficiency leads to an increase in PML and inflammatory cytokines, coherent with the notion that DNA replication and repair defects induce inflammatory responses. In feedback, increased IFN-β and ISG15 may result in resistance to cisplatin in BRCA2-deficient cells. The increase in IFNs and ISG15 upon Brca2 abrogation may be associated with telomere DNA damage, as MMC treatment in Brca2-proficient and telomerase-null cells led to an increase in *PML*, *IFN-β* and *ISG15* (Supplementary Figure S8). Of note, increased PML contributes to telomere LLPS and instigation of ALT-like telomere synthesis.

## Discussion

We have presented compelling evidence demonstrating that the loss of telomere G4 dynamicity is pivotal in inducing phase separation, a critical process for BIR-mediated telomere synthesis. Stabilized telomere G4 structures lead to the transcription of TERRA, triggered by TRCs, thereby facilitating the formation of TERRA-R-loops. These R-loops act as seeds for phase separation. Through the formation of liquid condensates, telomeres cluster together, facilitating the recruitment of proteins required for BIR and providing a platform for telomere synthesis in the absence of telomerase. Specifically, TERRA, extended from R-loops, recruits PRC2, resulting in the tri-methylation of H3K27 and promoting BIR. PRC2 within the liquid condensates may also contribute to the production of R-loops, which are essential for APB maintenance (Figure 7D). The physiological impact of telomere LLPS, or APBs, in ALT cancer cells is significant, as the liquid condensate enhances the efficiency of telomere recombination both *in cis* and *in trans*.

We demonstrated that BRCA2 deficiency can induce ALT through the loss of telomere G4 dynamicity. We showed that stable telomere G4 induces TERRA-R-loops, which instigate phase separation that plays a crucial role in this ALT-like telomere synthesis. Moreover, it is noteworthy that this phenomenon was observed in both mouse and human cells devoid of telomerase, consistent with the finding that telomeres undergoing telomere synthesis are marked with H3K27me3 in both mouse and human cells. However, this mechanism is not limited to BRCA2 disruption, as evidenced by findings from the U2OS ALT cancer cell line. Instead, it can be extended to as yet unidentified molecules that are involved in the maintenance of telomere G4 homeostasis. Thus, we have uncovered one pathway through which ALT originates: the disruption of telomere G4 homeostasis during replication.

LLPS has emerged as a crucial mechanism in numerous cellular processes, encompassing transcription, protein synthesis, signal transduction and DNA repair. Additionally, LLPS has been implicated in the pathogenesis of various diseases, including neurological disorders (52). However, whether the formation of liquid condensates is the cause or the result of the mentioned physiological functions remained unclear. In this study, we have demonstrated that the phase separation property is functionally crucial for maintaining telomere synthesis in ALT or ALT-like cells. This was evidenced by the fact that artificially induced phase separation was sufficient to substitute telomere synthesis activity in the absence of PML.

Throughout the study, we observed that BRCA2 abrogation induced telomere G4 stabilization, akin to treatment with the G4 stabilizer PDS. However, significant elevations in TERRA, R-loop, and APB assembly were observed only when the telomerase RNA component, *Terc*, was also abrogated. It is worth noting that telomere shortening has been linked to increased TERRA expression (53). When synergized with TERRA transcription in short telomeres, telomere G4 stabilization due to the loss of BRCA2 may lead to the generation of a sufficient level of TERRA-R-loops, thereby instigating assembly of the liquid condensates.

Interestingly, the PRC2 methyltransferase complex is recruited to ALT telomeres through association with TERRA protruding from telomere R-loops. Notably, >5 guanine-rich TERRA RNA molecules were required for PRC2 binding. This suggests that secondary structures, such as RNA G4, might play a role in recruiting PRC2. However, most endogenous RNAs remain unfolded due to abundant RNA-binding proteins (54). It was reported that TERRA regulates DNA G4 formation (55). Taken together, it is possible that TERRA-R-loops generated from BRCA2-deficient telomere G4 may, in turn, synergize to stabilize telomere DNA G4, exacerbating TERRA-R-loop generation and LLPS maintenance. Nevertheless, our *in vitro* experimental system demonstrated that a certain length of TERRA extending from R-loops is required for PRC2 association.

Similar to *Drosophila*PREs, PRC2 recruited by TERRA may contribute to R-loop accumulation at telomeres in ALT-like cells. Elimination of R-loops by ectopic expression of *RNH1* abolished telomere LLPS, which was restored upon LLPS engineering with TRF1-IDR. This suggests that R-loops initiate the assembly of telomere liquid condensates, and PRC2, recruited by TERRA-R-loops, contributes to the maintenance of LLPS by promoting R-loop formation through RNA–DNA strand exchange (45). Collectively, TERRA-R-loops initiate and maintain telomere phase separation, which is critical for ALT-like telomere synthesis. It was reported that TERRA initiates BIR in human ALT cells (56), and that the level of TERRA-R-loops plays a major role in telomere maintenance in ALT tumors (57). Our discovery that TERRA-R-loop-induced phase separation plays a pivotal role in ALT telomere synthesis aligns with the findings from the mentioned reports and represents a significant advancement in our current understanding of ALT mechanisms.

The co-treatment with the G4 stabilizer PDS and the EZH2-specific inhibitor EPZ-6438 selectively killed BRCA2-depleted ALT-like fibroblasts, suggesting that perturbing the mechanisms underlying telomere G4 and phase separation may be a promising approach for the treatment of ALT cancers. Silva and colleagues have suggested that TERRA transcription may be a versatile target for ALT cancer therapy, as TERRA increases ALT features and rapid telomere DNA loss (58). Targeting TERRA will also eliminate telomere LLPS, thereby interfering with TERRA transcription, or telomere R-loop formation may be a selective approach for targeting ALT cancers with APBs. Collectively, the mechanism uncovered in this study opens up new avenues for the treatment of ALT cancers with APBs, including BRCA2-deficient ALT-like cancers.

## Supplementary Material

gkae251_Supplemental_File

## Data Availability

The data underlying this article are available in the article and in its online supplementary data. Further data supporting the findings of this study are available from the corresponding authors upon reasonable request.
