# Supplementary material for: Disruption of G-quadruplex dynamicity by BRCA2 abrogation instigates phase separation and break-induced replication at telomeres
Source: Nucleic Acids Res. 2024 Apr 8;52(10):5756–73. doi: 10.1093/nar/gkae251 (PMC11162766; doi:10.1093/nar/gkae251)
Supplement: gkae251_Supplemental_File [file gkae251_supplemental_file.pdf]

Disruption of G-quadruplex dynamicity upon BRCA2 Abrogation instigates Phase separation and Break-induced Replication at telomeres

Jennifer J. Lee[1], *Hyungmin Kim*[1], *Haemin Park*[1], *UkJin Lee*[1], *Chaelim Kim*[3], *Min Lee*[3], *Yongdae Shin*[2, 3], *Ji-Jung Jung*[4], *Han-Byoel Lee*[4, 5, 6], *Wonshik Han*[4, 5, 6], *and Hyunsook Lee*[1]*

[1]Department of Biological Sciences & Institute of Molecular Biology and Genetics (IMBG), Seoul National University, 1 Gwanak-Ro, Gwanak-Gu, Seoul 08826, Korea

[2]Department of Mechanical Engineering, Seoul National University, Seoul 08826, Korea

[3]Interdisciplinary Program in Bioengineering, Seoul National University, Seoul 08826, Korea.

[4]Department of Surgery, Seoul National University College of Medicine, Seoul 03080, Korea

[5]Biomedical Research Institute, Seoul National University Hospital, Seoul 03080, Korea

[6]Cancer Research Institute, Seoul National University, Seoul 03080, Korea

* To whom correspondence should be addressed. Email: HL212@snu.ac.kr

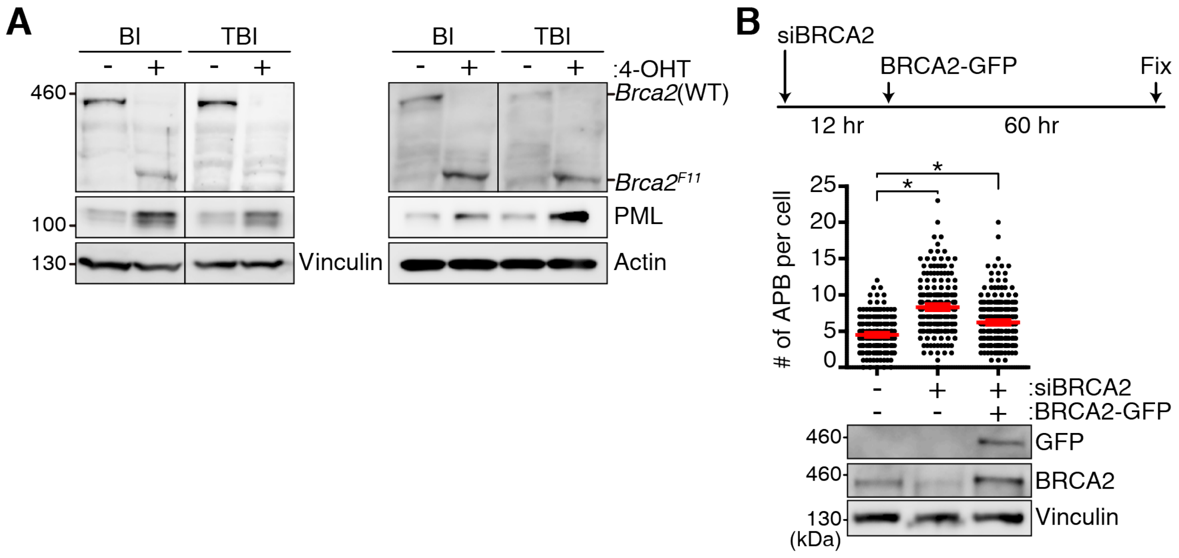

Supplementary Figure 1. Effect of BRCA2 abrogation in PML expression and APB assembly. (**A**) Western blot analysis of PML upon Brca2 depletion. BI and TBI fibroblasts from two different mouse each were treated with tamoxifen (4-OHT) and subjected to WB after four days. Note the abrogation of Brca2 and generation of *Brca2*^{F11} product upon 4-OHT treatment. (**B**) HeLa LT *TERC* KO cells were transfected with *siLuc* (control) or *siBRCA2* for 12hrs, followed by transfection with *GFP*- or siRNA-resistant *BRCA2-GFP*-expressing constructs. Scoring of APB and WB were conducted 72hrs post siRNA transfection. (Top) Number of APB per cell. >250 cells were analyzed each. * $p<0.0001$, Student's *t*-test (Mean ± SEM). (Bottom) Western blot analysis to assess knockdown of BRCA2 upon siRNA transfection and ectopic expression of *BRCA2-GFP*. WB with anti-GFP antibody confirmed the expression of siRNA-resistant *BRCA2-GFP* expression. WB with anti-BRCA2 antibody confirmed the depletion of endogenous BRCA2 and ectopic expression of *BRCA2-GFP*. Same blot was re-probed with anti-Vinculin antibody for normalization.

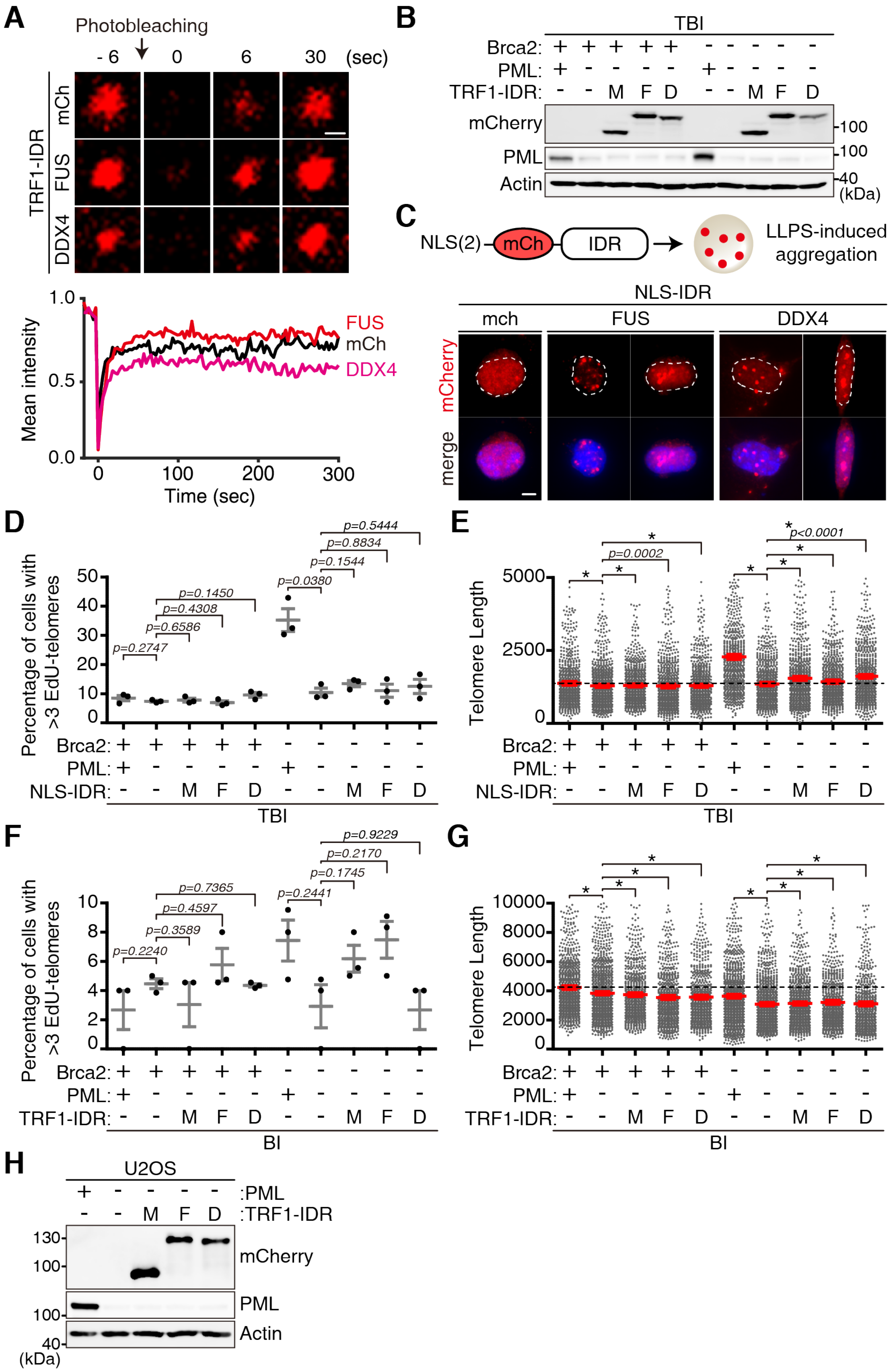
A
Photobleaching
- 6
0
6
30
(sec)
TRF1-IDR
mCh
FUS
DDX4
Mean intensity
1.0
0.5
0.0
0
100
200
300
Time (sec)
FUS
mCh
DDX4
B
TBI
Brca2:
PML:
TRF1-IDR:
mCherry
PML
Actin
100
40
(kDa)
C
NLS(2)
mCh
IDR
LLPS-induced aggregation
NLS-IDR
mch
FUS
DDX4
mCherry
merge
D
Percentage of cells with >3 EdU-telomeres
p=0.2747
p=0.6586
p=0.4308
p=0.1450
p=0.0380
p=0.1544
p=0.8834
p=0.5444
Brca2:
PML:
NLS-IDR:
TBI
E
Telomere Length
5000
2500
p=0.0002
p<0.0001
Brca2:
PML:
NLS-IDR:
TBI
F
Percentage of cells with >3 EdU-telomeres
p=0.2240
p=0.3589
p=0.4597
p=0.7365
p=0.2441
p=0.1745
p=0.2170
p=0.9229
Brca2:
PML:
TRF1-IDR:
BI
G
Telomere Length
10000
8000
6000
4000
2000
Brca2:
PML:
TRF1-IDR:
BI
H
U2OS
:PML
:TRF1-IDR
130
100
mCherry
PML
Actin
40
(kDa)

Supplementary Figure 2. Effect of engineered phase separation in break-induced replication (BIR). (**A**) (Top) Representative images of fluorescence recovery of TRF1-IDR-localized telomeres after photobleaching (FRAP). Scale bar, 0.5 µm. (Bottom) FRAP recovery curves, measured by mean fluorescent intensities in arbitrary units. Black, TRF1-mCh; red, TRF1-FUS; pink, TRF1-DDX4. (**B**) Western blot analysis: PML protein was depleted via siRNA transfection; the expression of TRF1-mCh (M), TRF1-FUS (F), TRF1-DDX4 (D) was detected by anti-mCherry antibody. (**C**) (Top) Illustration of control construct lacking TRF1. Two *NLS* (Nuclear localization signal) from SV40 are attached at the N-terminus of *mCherry*. (Bottom) Representative mCherry fluorescent images of each *NLS-IDR* expression in nucleus. Note the random nuclear aggregation. Scale bar, 5 µm. (**D-G**) Telomerase-positive BI and telomerase-negative TBI fibroblasts were subjected to G2 telomere synthesis assay (**D, F**) and telomere length measurement (**E, G**) in the presence (+) or absence (-) of Brca2. Depletion of PML and/or lentiviral transduction of M, F, D, respectively, were performed prior to the assay. Transfection of *siLuc* served as control. (**D, F**) Percentage of cells with more than three EdU-positive telomeres. >100 cells each were scored. (**E, G**) For T-FISH, >800 telomere foci from >50 cells each were scored. (**H**) Western blot analysis in U2OS cells: mCherry protein level after *TRF1-IDR* transduction; efficiency of PML knock-down after *siPML* transfection. All results are from three independent experiments. * $p<0.0001$, Student's *t*-test (Mean ± SEM).

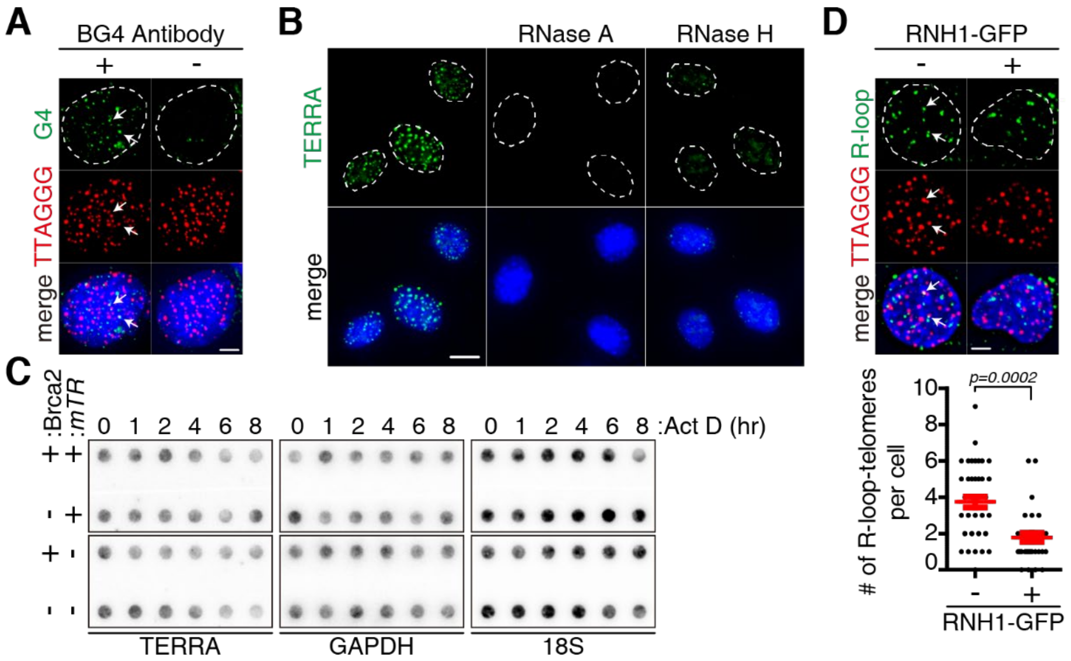

Supplementary Figure 3. Assessment of detecting telomere G4, R-loop and TERRA. (**A**) Validation of G4 and R-loop-specific antibodies in immunofluorescence. Representative images of G4. G4 was detected with immunofluorescence towards monoclonal antibody to FLAG (M2, Green), as the G4-specific antibody BG4 was tagged with FLAG (Merck Millipore). Presence of BG4 (+) detects G4, however anti-FLAG only (-) fails to detect G4. Telomeres (red) were labeled with T-FISH, with or without BG4 in Brca2-depleted TBI fibroblasts. White arrow, G4-positive telomere. Scale bar, 5 µm. (**B**) Assessing RNA-FISH in detecting *TERRA*. Representative images of *TERRA* RNA (green) in Brca2-depleted TBI fibroblasts. Recombinant RNase A or RNase H was treated for 1hr at 37°C prior to RNA-FISH. Scale bar, 10 µm. (**C**) Measurement of TERRA half-life. BI (top) and TBI (bottom) fibroblasts were treated with tamoxifen to deplete Brca2. Four days later, cells were treated with 10 µg/ml Actinomycin D (Act D) for 0 to 8hrs. Total RNA was extracted, dot-blotted on membrane, then hybridized with *TERRA*, *GAPDH*, or 18s rRNA probes. (**D**) (Top) Representative images of R-loop (green) and telomeres (red). Immunofluorescence with monoclonal R-loop-specific monoclonal antibody S9.6, coupled with T-FISH (red) was performed, with and without *RNH1-GFP* transduction in Brca2-depleted TBI fibroblasts. White arrow, R-loop-positive telomere. (Bottom) Number of R-loop-positive telomeres per cell. 100 cells each was counted. Scale bar, 5 µm. Student's *t*-test (Mean ± SEM).

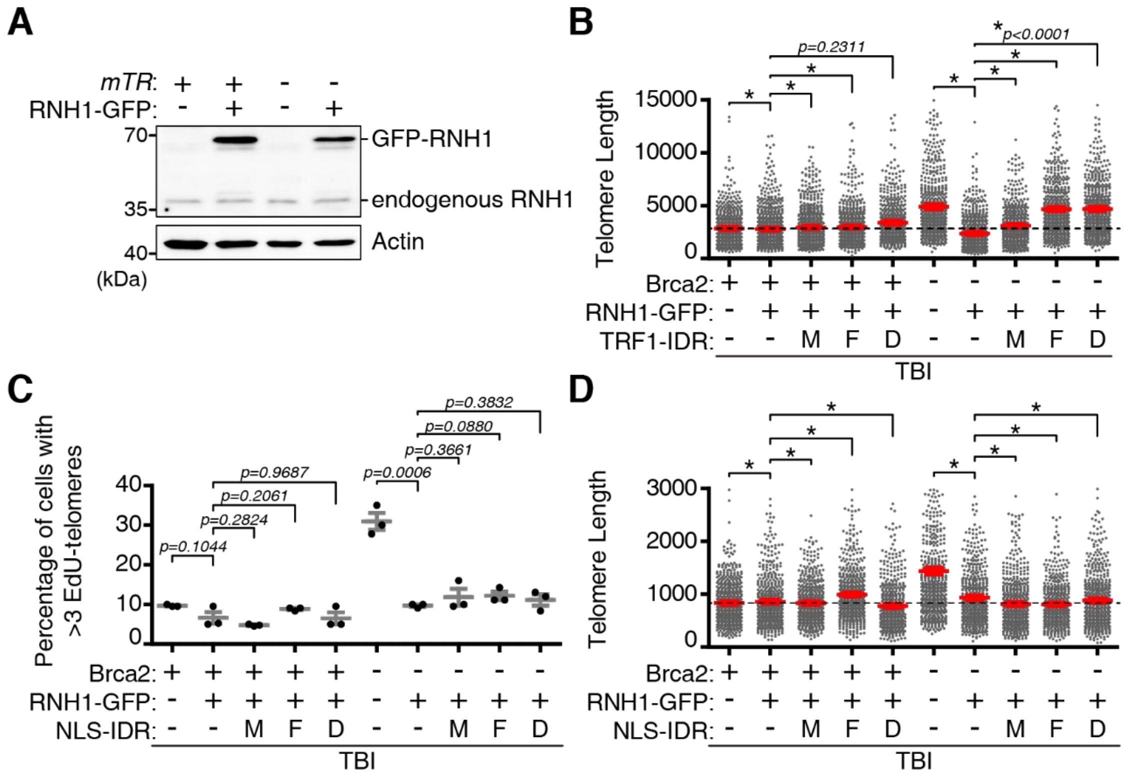

Supplementary Figure 4. R-loop is essential in Brca2-deficiency-induced ALT-like telomere synthesis. (**A-D**) Experiments were performed in *RNH1-GFP*-expressing TBI fibroblasts. Cells were infected with indicated lentivirus, M, F, D, respectively, in the presence (+) or absence (-) of Brca2. (**A**) Western blot analysis using anti-RNH1 antibody. Same blot was re-probed with anti-Actin antibody for normalization. (**B, D**) Telomere lengths were scored by the arbitrary fluorescence units of T-FISH. >700 telomere foci from >40 cells each were measured. (**C**) Percentage of cells with more than three EdU-positive telomeres. The results are from three independent experiments. >110 cells each were scored. * $p<0.0001$, Student's *t*-test (Mean ± SEM).

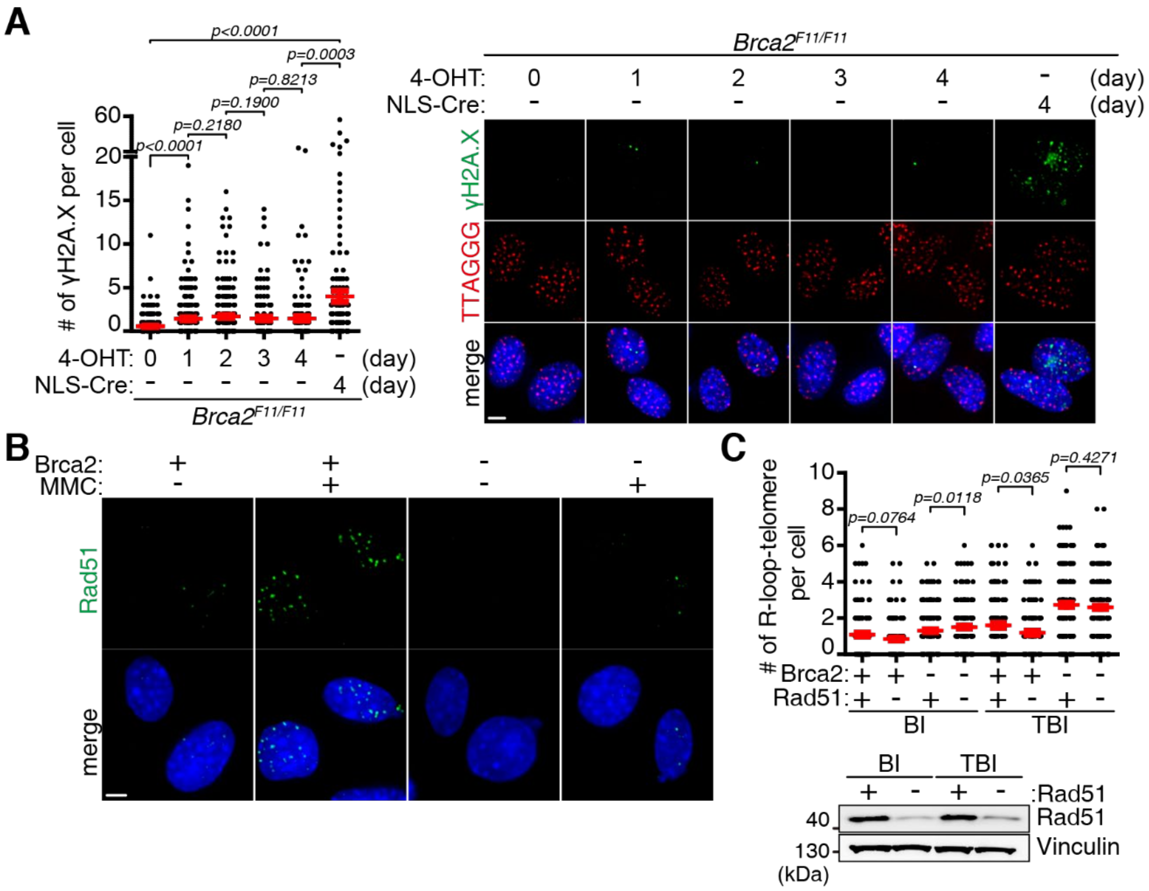

Supplementary Figure 5. Effect of Rad51 in R-loop formation in the presence or absence of Brca2. (**A**) Assessing cellular damage by tamoxifen treatment. Number of γ-H2A.X foci per cell was counted in wild-type *Brca2*$^{F11/F11}$ primary MEFs after treatment with 1 µM tamoxifen (4-OHT) for four days. For control, *Brca2*$^{F11/F11}$ MEFs were introduced with *NLS-Cre*-expressing construct and cultured for four days. Cells were fixed and immunostained with anti-γ-H2A.X antibody. Scale bar, 5 µm. >200 cells each were scored. (**B**) Brca2 is required for Rad51 to localize to damaged loci. Rad51 foci (green) was assessed upon treatment with mitomycin C (MMC) in the presence or absence of Brca2. Cells were exposed to 10 µM of MMC for 2hrs, then washed and released into drug-free medium for 5hrs before analysis. Scale bar, 5 µm. (**C**) Effect of Rad51 in telomeric R-loop formation. Telomerase-positive (BI) and telomerase-negative (TBI) fibroblasts were transduced with *shRad51*-expressing lentivirus, then treated with tamoxifen. Cells were immunostained with R-loop antibody (S9.6), followed by telomere FISH. The number of R-loop-positive telomeres was scored. >250 cells each were counted. (Bottom) WB analysis to assess Rad51 depletion. All experiments were three times independently. Student's *t*-test (Mean ± SEM).

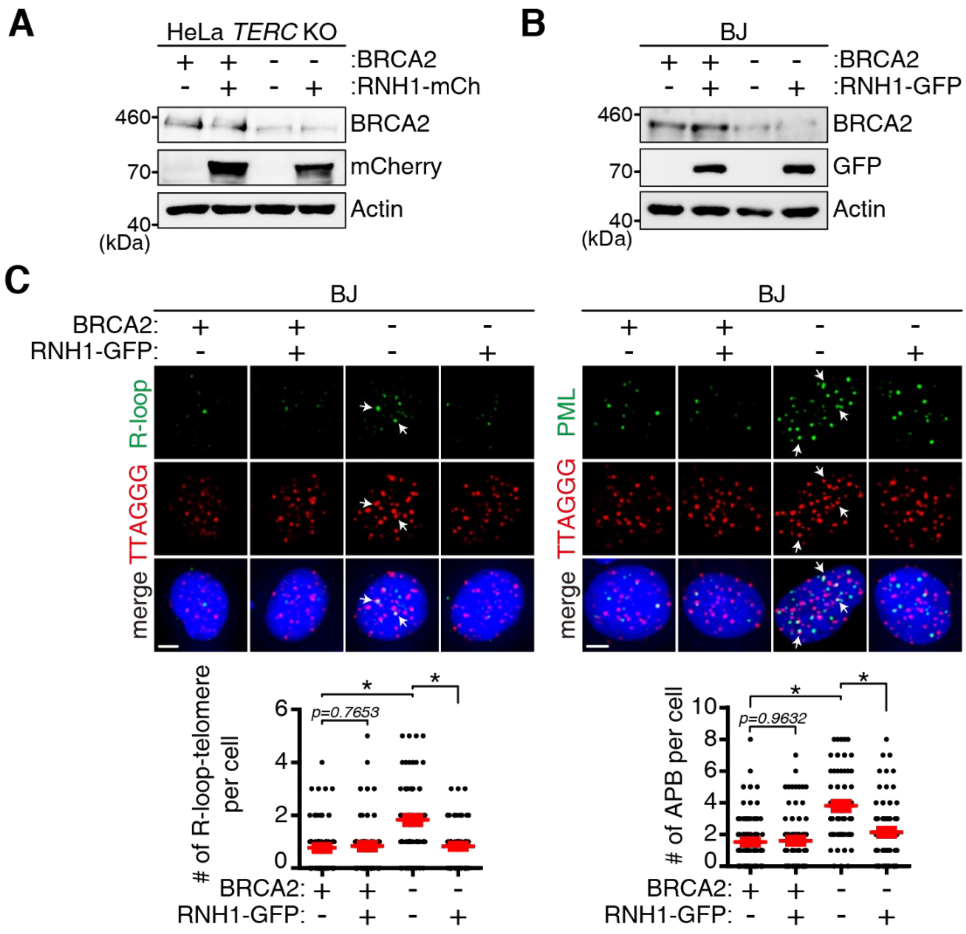

Supplementary Figure 6. Abrogation of BRCA2 in human ALT-like cells results in increase of R-loop and telomere LLPS. (**A**) Western blot analysis showing knock-down of BRCA2 post *siBRCA2* transfection and mCherry level after transfection of constructs expressing *RNH1-mcherry* in HeLa LT *TERC* KO cells. (**B-C**) BJ cells were transfected with *siLuc* (control) or *siBRCA2*, then transduced with *RNH1-GFP*-expressing lentivirus. (**B**) Western blot analysis with indicated antibodies. (**C**) (Top) Representative images of R-loop (green) and telomere (red) colocalization. White arrow, R-loop-positive telomere. Representative images of PML (green) and telomere (red) colocalization. White arrow, APB. Scale bar, 2.5 µm. (Bottom) Quantification of R-loop-positive telomeres and APBs in BJ cells. >200 cells each was counted. * $p<0.0001$, Student's *t*-test (Mean ± SEM).

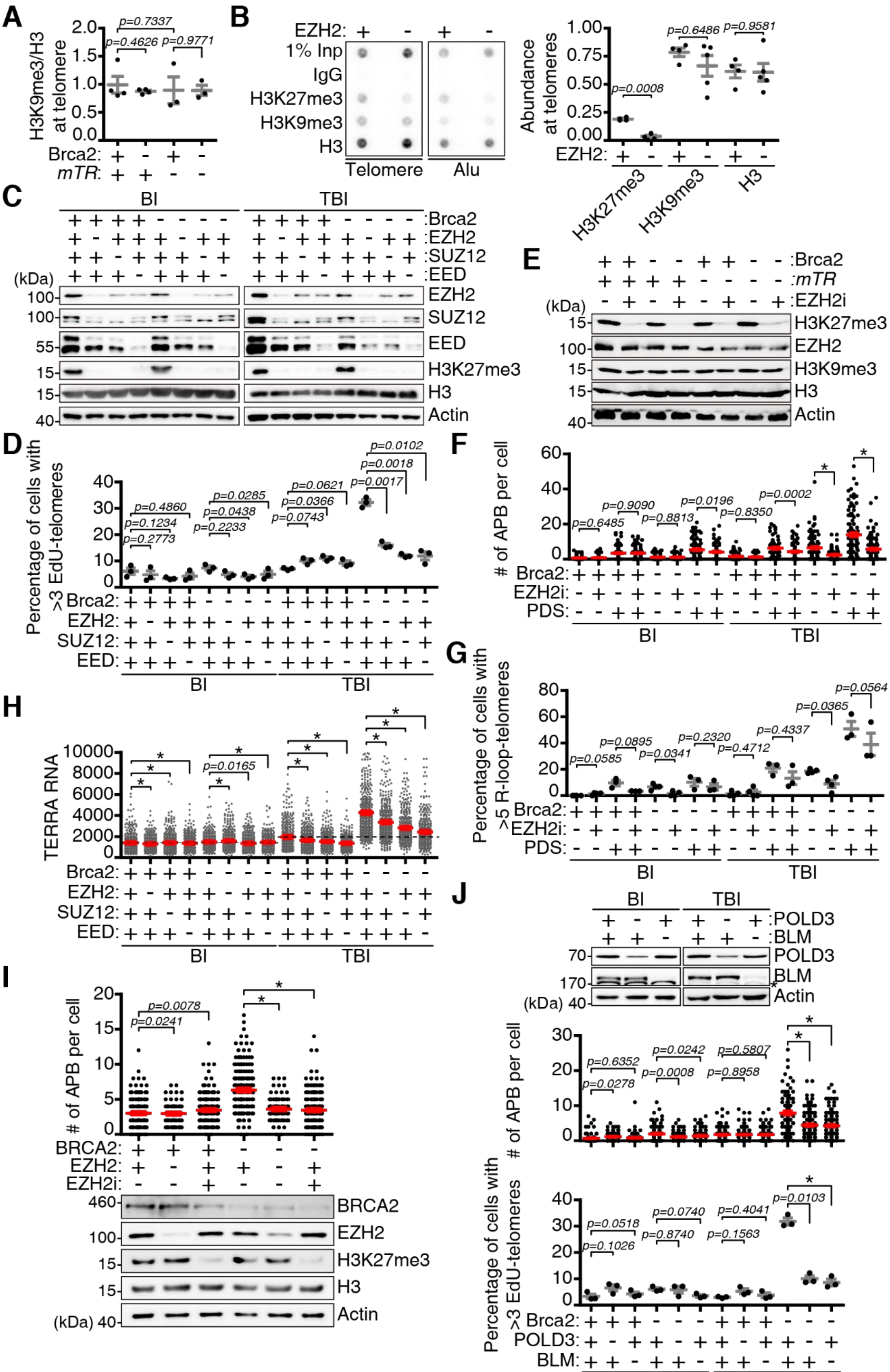
A
H3K9me3/H3
at telomere
p=0.7337
p=0.4626
p=0.9771
Brca2:
mTR:
B
EZH2:
1% Inp
IgG
H3K27me3
H3K9me3
H3
Telomere
Alu
Abundance
at telomeres
p=0.6486
p=0.9581
p=0.0008
EZH2:
H3K27me3
H3K9me3
H3
C
BI
TBI
:Brca2
:EZH2
:SUZ12
:EED
(kDa)
EZH2
SUZ12
EED
H3K27me3
H3
Actin
E
:Brca2
:mTR
:EZH2i
(kDa)
H3K27me3
EZH2
H3K9me3
H3
Actin
D
Percentage of cells with
>3 EdU-telomeres
p=0.4860
p=0.1234
p=0.2773
p=0.0285
p=0.0438
p=0.2233
p=0.0621
p=0.0366
p=0.0743
p=0.0102
p=0.0018
p=0.0017
Brca2:
EZH2:
SUZ12:
EED:
BI
TBI
F
# of APB per cell
p=0.6485
p=0.9090
p=0.8813
p=0.0196
p=0.8350
p=0.0002
Brca2:
EZH2i:
PDS:
BI
TBI
G
Percentage of cells with
>5 R-loop-telomeres
p=0.0585
p=0.0895
p=0.0341
p=0.2320
p=0.4712
p=0.4337
p=0.0365
p=0.0564
Brca2:
EZH2i:
PDS:
BI
TBI
H
TERRA RNA
p=0.0165
Brca2:
EZH2:
SUZ12:
EED:
BI
TBI
I
# of APB per cell
p=0.0078
p=0.0241
BRCA2:
EZH2:
EZH2i:
BRCA2
EZH2
H3K27me3
H3
Actin
(kDa)
J
BI
TBI
:POLD3
:BLM
POLD3
BLM
Actin
(kDa)
# of APB per cell
p=0.6352
p=0.0278
p=0.0242
p=0.0008
p=0.5807
p=0.8958
Percentage of cells with
>3 EdU-telomeres
p=0.0518
p=0.1026
p=0.0740
p=0.8740
p=0.4041
p=0.1563
p=0.0103
Brca2:
POLD3:
BLM:
BI
TBI

Supplementary Figure 7. Effect of PRC2 inhibition in phase separation and telomere synthesis. (**A**) Abundance of H3K9me3 at telomeres, normalized to H3, in Figure 5A. (**B**) Wild-type MEFs were transfected with siRNA targeting *EZH2*. (Left) Chromatin IP showing the level of H3K27me3 and H3K9me3 before and after *siEZH2* transfection. (Right) Abundance of H3K27me3, H3K9me3 and H3 at telomeres, normalized to input. Experiments were repeated four times independently. (**C**) Western blot analysis with indicated antibodies in BI (left) and TBI (right) fibroblasts after siRNA transfection of EZH2, SUZ12, EED, respectively. (**D**) Effect of depleting PRC2 complex in telomere synthesis. Graph shows the fraction of cells with more than three EdU-positive telomeres. >100 cells each were counted. (**E**) BI and TBI fibroblasts were exposed to 5 μM of EZH2 inhibitor EPZ-6438 for 48hrs, then subjected to western blot analysis with indicated antibodies to assess the level. The same blot was re-probed with anti-Actin antibodies for normalization. (**F-G**) BI and TBI fibroblasts treated (-) or left untreated (+) with 4-OHT were exposed to 5 μM PDS for 24hrs and/or 5 μM EPZ-6438 for 48hrs prior to fixation. (**F**) Treatment of EZH2 inhibitor abolishes APB. >100 cells each was scored. (**G**) The effect of EZH2 inhibitor in R-loop level in the presence or absence of Brca2. Cells were treated with PDS or left untreated to assess the effect of G4 stabilization. >200 cells each were scored. Graph is the outcome of three independent experiments. (**H**) Abundance of TERRA RNA in PRC2-deficient fibroblasts. Intensity of TERRA RNA was measured and shown in arbitrary units. >500 TERRA foci from >30 cells in each condition were scored. (**I**) Effect of EZH2 depletion with siRNA or inhibition (EPZ-6438 treatment) in BJ fibroblasts in the presence or absence of BRCA2. BRCA2 depletion was done by siRNA transfection. (Top) Number of APBs assessed. >250 cells each were scored. (Bottom) Western blot analysis with indicated antibodies. (**J**) Depletion of POLD3 or BLM reduces ALT-like telomere synthesis. (Top) Western blot analysis in BI and TBI fibroblasts with indicated antibodies to assess the efficiency of POLD3 or BLM knock-down, respectively, with shRNA introduction. (Bottom) Fibroblasts were transduced with shRNA for *POLD3* or *BLM*, respectively, and the number of APB and the level of telomere EdU incorporation were assessed. >150 cells each was scored. Graphs are the result of three independent experiments. * $p<0.0001$, Student's *t*-test (Mean ± SEM).

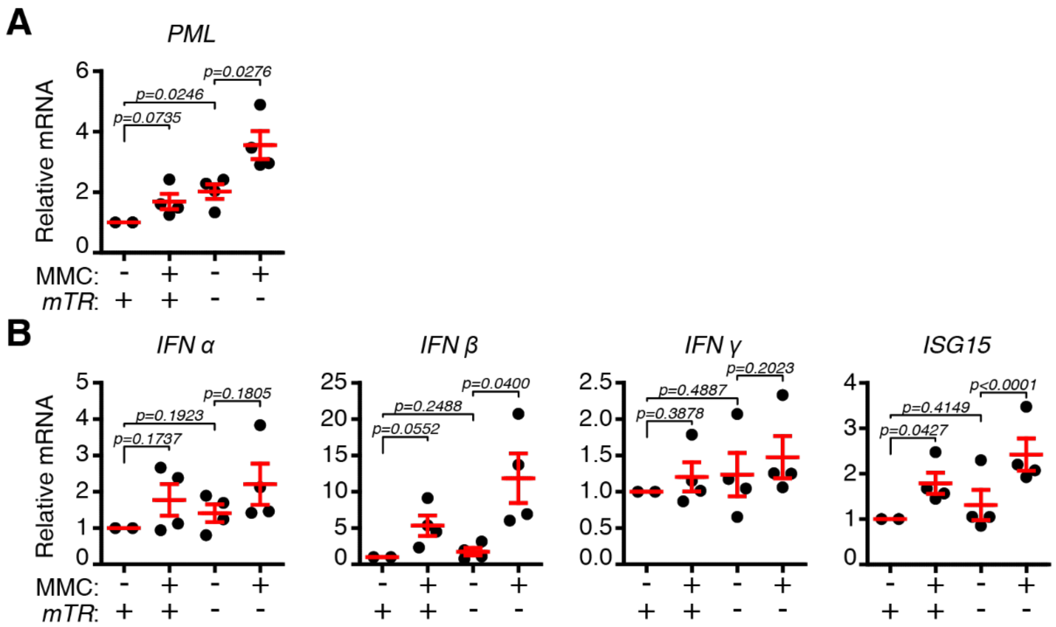

Supplementary Figure 8. Transcriptional changes upon mitomycin C (MMC) treatment. (**A-B**) Telomerase-positive BI (+*mTR*) and telomerase-negative TBI (-*mTR*) fibroblasts were treated with 2 μM MMC for two days, followed by RT-qPCR analysis of indicated genes. (**A**) Relative mRNA level of *PML*. (**B**) Relative mRNA level of *interferon-α, -β, -γ* and *ISG15*. Graph is the result of two independent experiments. Two technical replicates were employed for each PCR reaction. Student's t-test (Mean ± SEM).
